# Core components for effective infection prevention and control programmes: new WHO evidence-based recommendations

**DOI:** 10.1186/s13756-016-0149-9

**Published:** 2017-01-10

**Authors:** Julie Storr, Anthony Twyman, Walter Zingg, Nizam Damani, Claire Kilpatrick, Jacqui Reilly, Lesley Price, Matthias Egger, M. Lindsay Grayson, Edward Kelley, Benedetta Allegranzi, the WHO Guidelines Development Group, An Caluwaerts, Riham El-Asady, Dale Fisher, Petra Gastmeier, Alison Holmes, Kushlani Jayatilleke, Mary-Louise McLaws, Geeta Mehta, Shaheen Mehtar, Babacar Ndoye, Fernando Otaíza, Maria Clara Padoveze, Benjamin Park, Pierre Parneix, Didier Pittet, Valerie Robertson, Nanah Sesay-Kamara, Wing Hong Seto, Maha Talaat, Akeau Unahalekhaka, Evangelina Vazquez Curiel

**Affiliations:** 1 Infection Prevention and Control Global Unit, Service Delivery and Safety, HIS, World Health Organization, 20 Avenue Appia, 1211 Geneva 27, Switzerland; 2 Infection Control Programme, and WHO Collaborating Centre on Patient Safety, University of Geneva Hospitals and Faculty of Medicine, 4 Rue Gabrielle Perret-Gentil, 1211 Geneva 14, Switzerland; 3 Glasgow Caledonian University, Cowcaddens Road, Glasgow, G4 0BA UK; 4 Institute of Social and Preventive Medicine, University of Bern, Finkenhubelweg 11, 3012 Bern, Switzerland; 5 Austin Health and University of Melbourne, 145 Studley Road, PO Box 5555, Heidelberg, VIC Australia

**Keywords:** Infection prevention and control, HAI, IPC programmes, Hand hygiene, Antimicrobial resistance, IPC guideline, Surveillance, Multimodal strategy, IPC education, Workload, Staffing, Workforce, Bed occupancy, IPC practices, Universal health coverage

## Abstract

Health care-associated infections (HAI) are a major public health problem with a significant impact on morbidity, mortality and quality of life. They represent also an important economic burden to health systems worldwide. However, a large proportion of HAI are preventable through effective infection prevention and control (IPC) measures. Improvements in IPC at the national and facility level are critical for the successful containment of antimicrobial resistance and the prevention of HAI, including outbreaks of highly transmissible diseases through high quality care within the context of universal health coverage. Given the limited availability of IPC evidence-based guidance and standards, the World Health Organization (WHO) decided to prioritize the development of global recommendations on the core components of effective IPC programmes both at the national and acute health care facility level, based on systematic literature reviews and expert consensus. The aim of the guideline development process was to identify the evidence and evaluate its quality, consider patient values and preferences, resource implications, and the feasibility and acceptability of the recommendations. As a result, 11 recommendations and three good practice statements are presented here, including a summary of the supporting evidence, and form the substance of a new WHO IPC guideline.

**Electronic supplementary material:**

The online version of this article (doi:10.1186/s13756-016-0149-9) contains supplementary material, which is available to authorized users.

## Introduction

Infection prevention and control (IPC) is a universally relevant component of all health systems and affects the health and safety of both people who use health services and those who provide them. Health care-associated infections (HAI) are one of the most common adverse events in care delivery and both the endemic burden and epidemics are a major public health problem. In 2011, the World Health Organization (WHO) [1] reported that on average 7% of patients in developed and 15% in low- and middle-income countries (LMICs) suffer from at least one HAI at any given time, with attributable mortality estimated at 10% [2]. The burden of HAI is significantly higher in LMICs and affects especially high-risk populations, such as patients admitted to neonatal and intensive care units where the frequency of HAI is two to 20 times higher compared to high-income countries, notably for device-associated infections [2].

HAI has a significant and largely avoidable economic impact at both the patient and population levels, including out-of-pocket costs to patients and costs incurred through lost productivity due to morbidity and mortality. Although the evidence related to the economic burden of HAI is limited, particularly in LMICs, available data from the USA and Europe suggest costs estimated at several billions. According to the US Centers for Disease Control and Prevention, the overall annual direct medical costs of HAI to hospitals in the USA alone ranges from US$ 35.7 to 45 billion [3], while the annual economic impact in Europe is as high as € 7 billion [4].

Although significant progress has been made to reduce HAI in many parts of the world, a number of emerging events have underlined the need to support countries in the development and strengthening of IPC with the objective to achieve resilient health systems, both at the national and facility levels. In recent years, global public health emergencies of international concern, such as the Middle East respiratory syndrome coronavirus and the Ebola virus disease outbreaks, revealed gaps in IPC measures applied by the countries concerned. Furthermore, the current review of the International Health Regulations and the Global Action Plan to combat antimicrobial resistance (AMR) [5–9] called for strengthening IPC across nations. This will also contribute to achieve strategic goal 5 of the WHO Framework on integrated people-centred health services and the United Nations Sustainable Development Goals - in particular, those related to universal access to water and sanitation and hygiene (WASH), quality health service delivery in the context of universal health coverage, and the reduction of neonatal and maternal mortality.

In consideration of these factors, WHO decided to prioritize the development of evidence-based recommendations on the essential elements (“core components”) of IPC programmes at the national and facility level. With the exception of a set of IPC core components previously identified by experts during a WHO meeting [10], there is a major gap in international evidence-based recommendations as to what should constitute the key elements of effective IPC programmes at the national and facility level. A first step was made by a project initiated by the European Centre for Disease Prevention and Control, which identified key components for hospital organization, management and structure for the prevention of HAI based on evidence and expert consensus [11].

We present here the new WHO core components for IPC improvement to be implemented in acute health care facilities and at the national level (www.who.int/gpsc/ipc-components/en/), with a brief description of the background scientific evidence. This guidance builds on the initial momentum of the WHO IPC core components interim document published in 2009 [8]. The recommendations were elaborated according to the best available scientific evidence and expert consensus with the ultimate aim to ensure a high quality of health service delivery for every person accessing health care, as well as to protect the health workforce delivering those services.

The intended audience on a national level is primarily policy-makers responsible for establishing and monitoring national IPC programmes and delivering AMR National Action Plans. The recommendations are also relevant to those in charge of health facility accreditation/regulation, health care quality improvement, public health, disease control, WASH, occupational health, and antimicrobial stewardship programmes. At the facility level, the main target audience is facility-level administrators, IPC and WASH leaders and teams, safety and quality leads and managers, and regulatory bodies. Allied organizations will also have an interest in the core components, including academic institutions, national IPC professional bodies, nongovernmental organizations involved in IPC, and civil society groups.

## Methods

The WHO guidelines were developed according to the requirements described in the WHO *handbook for guideline development* [12]. The first source of evidence was the review published by the “Systematic review and evidence-based guidance on organization of hospital infection control programmes” (SIGHT) group [11], which included publications from 1996 to 2012. This review was updated to include literature published up to 23 November 2015. An additional systematic review with the same objectives was performed, but with a focus on the national level. Key research questions were identified and formulated according to the PICO (Population/Participants, Intervention, Comparator and Outcomes) process. In addition, an inventory of national and regional IPC action plans and strategic documents was undertaken as part of the background to these guidelines.

### Search strategy selection criteria and evidence assessment

We searched Medline (via EBSCO); the Excerpta Medica Database (EMBASE) (via Ovid); the Cumulative Index to Nursing and Allied Health Literature (CINAHL); the Cochrane Central Register of Controlled Trials (CENTRAL); the Outbreak Database; and the WHO Institutional Repository for Information Sharing. The time limit was between 1 January 2013 and 23 November 2015 for the update of the SIGHT review, and between 1 January 2000 and 31 December 2015 for the national level review. Studies in English, French, Portuguese and Spanish were eligible. A comprehensive list of search terms was used in both reviews, including Medical Subject Headings (MeSH) (Additional files 1 and 2). In the earlier review done by the SIGHT group, the quality of the evidence was assessed using the “Integrated quality Criteria for Review Of Multiple Study designs” (ICROMS) scoring system [13]. The SIGHT review update and the review focusing on the national level used the risk of bias criteria developed for the Cochrane Effective Practice and Organization of Care (EPOC) reviews [14]. Due to different methodologies and outcome measures, it was not possible to perform a meta-analysis for any of the reviews.

### Methods for the development of recommendations

The recommendations were developed by a panel of international experts based on the available evidence and its quality, the balance between benefits and harms, cost and resource implications, acceptability and feasibility, and user and patient values and preferences. Members of the panel were key international IPC experts and country delegates. Geographical and gender balance were ensured, including representation from various professional groups, such as physicians, nurses, clinical microbiologists, IPC and infectious disease specialists, epidemiologists, researchers, and patient representatives. The strength of recommendations was rated as either “strong” (the panel was confident that the benefits of the intervention outweighed the risks) or “conditional” (the panel considered that the benefits of the intervention probably outweighed the risks). In the absence of methodologically sound, direct evidence on the effectiveness of interventions, good practice statements were developed for IPC components that were judged essential by consensus [15]. The recommendations and their individual strength, the good practice statements, and the key remarks for implementation made by the panel are presented in Table 1.

**Table 1:** Summary of IPC core components and key remarks

Core component	Recommendation or good practice statement	Key remarks	Strength of recommendation and quality of evidence
1. IPC programmes	1a. The panel recommends that an IPC programme with a dedicated, trained team should be in place in each acute health care facility for the purpose of preventing HAI and combating AMR through IPC good practices.	• The organization of IPC programmes must have clearly defined objectives based on local epidemiology and priorities according to risk assessment and functions that align with and contribute to the prevention of HAI and the spread of AMR in health care. • It is critical for a functioning IPC programme to have dedicated, trained professionals in every acute care facility. A minimum ratio of one full-time or equivalent infection preventionist (nurse or doctor) per 250 beds should be available. However, there was a strong opinion that a higher ratio should be considered, for example, one infection preventionist per 100 beds, due to increasing patient acuity and complexity, as well as the multiple roles and responsibilities of the modern preventionist. • Good quality microbiological laboratory support is a very critical factor an effective IPC programme.	Strong, very low quality
	1b. Active, stand-alone, national IPC programmes with clearly defined objectives, functions and activities should be established for the purpose of preventing HAI and combating AMR through IPC good practices. National IPC programmes should be linked with other relevant national programmes and professional organizations.	• The organization of national IPC programmes must be established with clear objectives, functions, appointed infection preventionists and a defined scope of responsibilities. Minimum objectives should include: ▪ goals to be achieved for endemic and epidemic infections ▪ development of recommendations for IPC processes and practices that are known to be effective in preventing HAI and the spread of AMR • The IHR (2005) and the WHO Global Action Plan on AMR (2015) support national level action on IPC as a central part of health systems’ capacity building and preparedness. This includes the development of national plans for preventing HAI, the development or strengthening of national policies and standards of practice regarding IPC activities in health facilities, and the associated monitoring of the implementation of and adherence to these national policies and standards. • The organization of the programme should include (but not be limited to) at least the following components: ▪ appointed technical team of trained infection preventionists, including medical and nursing professionals ▪ the technical teams should have formal IPC training and allocated time according to tasks ▪ the team should have the authority to make decisions and to influence field implementation ▪ the team should have a protected and dedicated budget according to planned IPC activity and support by national authorities and leaders • The linkages between the national IPC programme and other related programmes are key and should be established and maintained. • An official multidisciplinary group, committee or an equivalent structure should be established to interact with the IPC technical team.	Good practice statement
2. IPC guidelines	The panel recommends that evidence-based guidelines should be developed and implemented for the purpose of reducing HAI and AMR. The education and training of relevant health care workers on the guideline recommendations and the monitoring of adherence with guideline recommendations should be undertaken to achieve successful implementation.	Health care facility • Appropriate IPC expertise is necessary to write or adapt and adopt a guideline both at the national and health care facility level. Guidelines should be evidence-based and reference international or national standards. Adaptation to local conditions should be considered for the most effective uptake and implementation. • Monitoring adherence to guideline implementation is essential. National level • Developing relevant evidence-based national IPC guidelines and related implementation strategies is one of the key functions of the national IPC programme. • The national IPC programme should also ensure that the necessary infrastructures and supplies to enable guideline implementation are in place. • The national IPC programme should support and mandate health care workers’ education and training focused on the guideline recommendations.	Strong, very low quality
3. IPC education and training	3a. The panel recommends that IPC education should be in place for all health care workers by utilizing team- and task-based strategies that are participatory and include bedside and simulation training to reduce the risk of HAI and AMR.	• IPC education and training should be a part of an overall health facility education strategy, including new employee orientation and the provision of continuous educational opportunities for existing staff, regardless of level and position (for example, including also senior administrative and housekeeping staff). • Three categories of human resources were identified as targets for IPC training and requiring different strategies and training contents: IPC specialists, all health care workers involved in service delivery and patient care, and other personnel that support health service delivery (administrative and managerial staff, auxiliary service staff, cleaners, etc.). • Periodic evaluations of both the effectiveness of training programmes and assessment of staff knowledge should be undertaken on a routine basis.	Strong, moderate quality
	3b. The national IPC programme should support the education and training of the health workforce as one of its core functions.	• The IPC national team plays a key role to support and make IPC training happen at the facility level. • To support the development and maintenance of a skilled, knowledgeable health workforce, national pregraduate and postgraduate IPC curricula should be developed in collaboration with local academic institutions. • In the curricula development process, it is advisable to refer to international curricula and networks for specialized IPC programmes and to adapt these documents and approaches to national needs and local available resources. • The national IPC programme should provide guidance and recommendations for in-service training to be rolled out at the facility level according to detailed IPC core competencies for health care workers and covering all professional categories listed in core component 3a.	Good practice statement
4. Surveillance	4a. The panel recommends that facility-based HAI surveillance should be performed to guide IPC interventions and detect outbreaks, including AMR surveillance with timely feedback of results to health care workers and stakeholders and through national networks.	• Surveillance of HAI is critical to inform and guide IPC strategies. • Health care facility surveillance should be based on national recommendations and standard definitions and customized to the facility according to available resources with clear objectives and strategies. Surveillance should provide information for: ▪ describing the status of infections associated with health care (that is, incidence and/or prevalence, type, aetiology and, ideally, data on severity and the attributable burden of disease). ▪ identification of the most relevant AMR patterns. ▪ identification of high risk populations, procedures and exposures. ▪ existence and functioning of WASH infrastructures, such as a water supply, toilets and health care waste disposal. ▪ early detection of clusters and outbreaks (that is, early warning system). ▪ Evaluation of the impact of interventions. • Quality microbiology and laboratory capacity is essential to enable reliable HAI surveillance. • The responsibility for planning and conducting surveillance and analysing, interpreting and disseminating the collected data remains usually with the IPC committee and the IPC team. • Methods for detecting infections should be active. Different surveillance strategies could include the use of prevalence or incidence studies. • Hospital-based infection surveillance systems should be linked to integrated public health infection surveillance systems. • Surveillance reports should be disseminated in a timely manner to those at the managerial or administration level (decision-makers) and the unit/ward level (frontline health care workers). • A system for surveillance data quality assessment is of the utmost importance.	Strong, very low quality
	4b. The panel recommends that national HAI surveillance programmes and networks that include mechanisms for timely data feedback and with the potential to be used for benchmarking purposes should be established to reduce HAI and AMR.	• National HAI surveillance systems feed in to general public health capacity building and the strengthening of essential public health functions. National surveillance programmes are also crucial for the early detection of some outbreaks in which cases are described by the identification of the pathogen concerned or a distinct AMR pattern. Furthermore, national microbiological data about HAI aetiology and resistance patterns also provide information relevant for policies on the use of antimicrobials and other AMR-related strategies and interventions. • Establishing a national HAI surveillance programme requires full support and engagement by governments and other respective authorities and the allocation of human and financial resources. • National surveillance should have clear objectives, a standardized set of case definitions, methods for detecting infections (numerators) and the exposed population (denominators), a process for the analysis of data and reports and a method for evaluating the quality of the data. • Clear regular reporting lines of HAI surveillance data from the local facility to the national level should be established. • International guidelines on HAI definitions are important, but it is the adaptation at country level that is critical for implementation. • Microbiology and laboratory capacity and quality are critical for national and hospital-based HAI and AMR surveillance. Standardized definitions and laboratory methods should be adopted. • Good quality microbiological support provided by at least one national reference laboratory is a critical factor for an effective national IPC surveillance programme. • A national training programme for performing surveillance should be established to ensure the appropriate and consistent application of national surveillance guidelines and corresponding implementation toolkits. • Surveillance data is needed to guide the development and implementation of effective control interventions.	Strong, very low quality
5. Multimodal strategies	5a. The panel recommends that IPC activities using multimodal strategies should be implemented to improve practices and reduce HAI and AMR.	• Successful multimodal interventions should be associated with an overall organizational culture change as effective IPC can be a reflector of quality care, a positive organizational culture and an enhanced patient safety climate. • Successful multimodal strategies include the involvement of champions or role models in several cases • Implementation of multimodal strategies within health care institutions needs to be linked with national quality aims and initiatives, including health care quality improvement initiatives or health facility accreditation bodies.	Strong, low quality
	5b. The panel recommends that national IPC programmes should coordinate and facilitate the implementation of IPC activities through multimodal strategies on a nationwide or subnational level.	• The national approach to coordinating and supporting local (health facility level) multimodal interventions should be within the mandate of the national IPC programme and be considered within the context of other quality improvement programmes or health facility accreditation bodies. • Ministry of health support and the necessary resources, including policies, regulations and tools, are essential for effective central coordination. This recommendation is to support facility level improvement. • Successful multimodal interventions should be associated with overall cross-organizational culture change as effective IPC can be a reflector of quality care, a positive organizational culture and an enhanced patient safety climate. • Strong consideration should be given to country adaptation of implementation strategies reported in the literature, as well as to feedback of results to key stakeholders and education and training to all relevant persons involved in the implementation of the multimodal approach.	Strong, low quality
6. Monitoring/audit of IPC practices and feedback	6a. The panel recommends that regular monitoring/audit and timely feedback of health care practices according to IPC standards should be performed to prevent and control HAI and AMR at the health care facility level. Feedback should be provided to all audited persons and relevant staff.	• The main purpose of auditing/monitoring practices and other indicators and feedback is to achieve behaviour change or other process modification to improve the quality of care and practice with the goal of reducing the risk of HAI and AMR spread. Monitoring and feedback are also aimed at engaging stakeholders, creating partnerships and developing working groups and networks. • Sharing the audit results and providing feedback not only with those being audited (individual change), but also with hospital management and senior administration (organizational change) are critical steps. IPC teams and committees (or quality of care committees) should also be included as IPC care practices are quality markers for these programmes. • IPC programmes should be periodically evaluated to assess the extent to which the objectives are met, the goals accomplished, whether the activities are being performed according to requirements and to identify aspects that may need improvement identified via standardized audits. Important information that may be used for this purpose includes the results of the assessment of compliance with IPC practices, other process indicators (for example, training activities), dedicated time by the IPC team and resource allocation.	Strong, low quality
	6b. The panel recommends that a national IPC monitoring and evaluation programme should be established to assess the extent to which standards are being met and activities are being performed according to the programme’s goals and objectives. Hand hygiene monitoring with feedback should be considered as a key performance indicator at the national level.	• Regular monitoring and evaluation provides a systematic method to document the progress and impact of national programmes in terms of defined indicators, for example, tracking hand hygiene improvement as a key indicator, including hand hygiene compliance monitoring. • National level monitoring and evaluation should have in place mechanisms that: ▪ Provide regular reports on the state of the national goals (outcomes and processes) and strategies. ▪ Regularly monitor and evaluate the WASH services, IPC activities and structure of the health care facilities through audits or other officially recognized means. ▪ Promote the evaluation of the performance of local IPC programmes in a non- punitive institutional culture.	Strong, moderate quality
7. Workload, staffing and bed occupancy (*acute health care facility only*)	The panel recommends that the following elements should be adhered to in order to reduce the risk of HAI and the spread of AMR: (1) bed occupancy should not exceed the standard capacity of the facility; (2) health care worker staffing levels should be adequately assigned according to patient workload.	• Standards for bed occupancy should be one patient per bed with adequate spacing between patient beds and that this should not be exceeded. • Intended capacity may vary from original designs and across facilities and countries. For these reasons, it was proposed that ward design regarding bed capacity should be adhered to and in accordance with standards. In exceptional circumstances where bed capacity is exceeded, hospital management should act to ensure appropriate staffing levels that meet patient demand and an adequate distance between beds. These principles apply to all units and departments with inpatient beds, including emergency departments. • The WHO Workload Indicators of Staffing Need method provides health managers with a systematic way to determine how many health workers of a particular type are required to cope with the workload of a given health facility and decision-making (http://www.who.int/hrh/resources/wisn_user_manual/en/). • Overcrowding was recognized as being a public health issue that can lead to disease transmission.	Strong, very low quality
8. Built environment, materials and equipment for IPC at the facility level (*acute health care facility only*)	8a. Patient care activities should be undertaken in a clean and/or hygienic environment that facilitates practices related to the prevention and control of HAI, as well as AMR, including all elements around the WASH infrastructure and services and the availability of appropriate IPC materials and equipment.	• An appropriate environment, WASH services and materials and equipment for IPC are a core component of effective IPC programmes at health care facilities. • Ensuring an adequate hygienic environment is the responsibility of senior facility managers and local authorities. However, the central government and national IPC and WASH programmes also play an important role in developing standards and recommending their implementation regarding adequate WASH services in health care facilities, the hygienic environment, and the availability of IPC materials and equipment at the point of care. • WHO standards for drinking water quality, sanitation and environmental health in health care facilities should be implemented.	Good practice statement
	8b. The panel recommends that materials and equipment to perform appropriate hand hygiene should be readily available at the point of care.	• WHO standards for the adequate number and appropriate position of hand hygiene facilities should be implemented in all health care facilities.	Strong, very low quality

*HAI* health care-associated infection, *AMR* antimicrobial resistance, *IPC* infection prevention and control, *IHR* International Health Regulations, *WASH* water, sanitation and health, *NA* not applicable

## Core component 1: IPC programmes

IPC programmes are one component of safe, high-quality health service delivery. A WHO global survey published in 2015 revealed major weaknesses in national IPC capacity [16]. Among the 133 respondent countries, only 54 had a national IPC programme (41%) in place and even fewer reported a programme in all tertiary hospitals (39/133; 29%). In addition, our inventory of IPC national strategies or action plans showed that while the vast majority of documents (85%) across all regions addressed IPC programme structure and goals, only 60% specified the importance of having qualified and dedicated staff to support the programme, and only 44% highlighted the need for an adequate budget and WASH infrastructure.

### Acute health care facility level

#### Recommendation

The panel recommends that an IPC programme with a dedicated, trained team should be in place in each acute health care facility for the purpose of preventing HAI and combating AMR through IPC good practices.

***(Strong recommendation, very low quality of evidence)***

Evaluation of the evidence from two studies (one controlled before-after study [17] and one interrupted time series [18]) showed that IPC programmes including dedicated, trained professionals are effective in reducing HAI in acute care facilities. Despite the limited published evidence and its very low quality, the panel strongly recommended that an IPC programme should be in place in all acute health care facilities. This decision was based on the large effect of HAI reduction reported in the two studies and on the panel’s conviction that the existence of an IPC programme is the necessary premise for any IPC action.

### National level

#### Good practice statement

The panel supports the establishment of stand-alone, active national IPC programmes with clearly defined objectives, functions and activities for the purpose of preventing HAI and combating AMR through IPC good practices. National IPC programmes should be linked to other relevant national programmes and professional organizations.

Several studies concerning the implementation of nationwide multimodal programmes aimed at reducing specific types of infections were retrieved, e.g. catheter-associated bloodstream infection. However, no evidence was available to evaluate the effectiveness of a more comprehensive national IPC programme and, therefore to formulate a recommendation. Despite this, experts and country representatives brought very clear examples where an active and sustained national IPC programme with effectively implemented plans has led to improvement of national HAI rates and/or the reduction of infections due to multidrug-resistant organisms. In addition, the International Health Regulations (2005) [8] and the WHO Global Action Plan on AMR (2015) [9] support national level action on IPC as a central part of health systems’ capacity building and preparedness. This includes the development of national plans for preventing HAI, the development or strengthening of national policies and standards of practice regarding IPC activities in health care facilities, and the associated monitoring of the implementation of and adherence to these national policies and standards. Therefore, the panel strongly affirmed that each country should have a stand-alone, active national IPC programme to prevent HAI, to combat AMR through IPC good practices, and thus to ultimately achieve safe, high-quality health service delivery.

## Core component 2: IPC guidelines

The availability of technical guidelines consistent with the available evidence is essential to provide a robust framework to support the performance of good practices. Importantly, the existence of guidelines alone is not sufficient to ensure their adoption and implementation science principles and findings clearly indicate that local adaptation is a prerequisite for successful guideline adoption. The WHO inventory identified that on average, 74% of national IPC documents addressed the development, dissemination, and implementation of technical guidelines and 43% emphasized the importance of local adaptation. Over 80% of national documents addressed the need for the training of all staff in IPC measures.

### National and acute health care facility level

#### Recommendation

The panel recommends that evidence-based guidelines should be developed and implemented for the purpose of reducing HAI and AMR. The education and training of relevant health care workers on the guideline recommendations and the monitoring of adherence with guideline recommendations should be undertaken to achieve successful implementation.

***(Strong recommendation, very low quality of evidence)***

Evaluation of the evidence from six studies (three non-controlled before-after studies [19–21], one non-controlled interrupted time series [22] and two qualitative studies [23, 24]) showed that guidelines on the most important IPC good practices and procedures are effective to reduce HAI when implemented in combination with health care workers’ education and training. Three reports were from an upper-middle-income country (Argentina) [20–22] and the remaining ones were from the USA [19, 23, 24]. The overall quality of evidence was very low. However, the panel unanimously decided to strongly recommend the development and implementation of IPC guidelines, supported by health care workers’ education and training and monitoring of adherence to guidelines.

## Core component 3: IPC education and training

IPC education spans all domains of health service delivery and is relevant to all health care workers, ranging from frontline workers to administrative management. Our inventory of IPC national strategies or action plans revealed that the vast majority of documents (81%) across all regions highlighted the importance of building basic IPC knowledge among all health care workers. However, only 51% also addressed specialized training of IPC professionals, and only 37% specified that specialized staff responsible for IPC are needed at the facility level.

### Acute health care facility level

#### Recommendation

The panel recommends that IPC education should be in place for all health care workers by utilizing team- and task-based strategies that are participatory and include bedside and simulation training to reduce the risk of HAI and AMR.

***(Strong recommendation, moderate quality of evidence)***

Evaluation of the evidence from 15 studies (five interrupted case series [25–29], five qualitative [24, 30–33], two controlled before-after [34, 35], two non-controlled before-after [36, 37], and one mixed methods [38]) showed that IPC education that involves frontline health care workers in a practical, hands-on approach and incorporates individual experiences is associated with decreased HAI and increased hand hygiene compliance. Twelve studies were from high-income countries [24–28, 31–34, 36–38], two from one upper-middle-income country [29, 35], and one from a LMIC [30]. The overall quality of evidence was moderate. As a result, the panel decided to strongly recommend that IPC education and training should be in place for all health care workers using a team- and task-oriented approach.

### National level

#### Good practice statement

The national IPC programme should support the education and training of the health workforce as one of its core functions.

Several studies related to the implementation of nationwide multimodal programmes were retrieved (see Core component 5). These included a strong health care worker education and training component with the aim to reduce specific types of infections, e.g. catheter-associated bloodstream infections. In addition, health care worker training was found to be an essential component for effective guideline implementation (see Core component 2). However, there was no specific evidence on the effectiveness of national curricula or IPC education and training per se. Our inventory highlighted that training for all health care workers was a strong feature of existing national IPC documents. This ranged from 57% of documents in the WHO European Region to 100% in the African Region. Therefore, the panel considered that it was important to develop a good practice statement to recommend that IPC national programmes should support education and training of the health workforce as one of its core functions to prevent HAIs and AMR and to achieve safe, high-quality health service delivery.

## Core component 4: HAI surveillance

It is widely acknowledged that surveillance systems allow the evaluation of the local burden of HAI and AMR and contribute to the early detection of HAI and new patterns of AMR, including the identification of clusters and outbreaks. IPC activities should respond to the actual needs of the health care facility, based on the local HAI situation and compliance with IPC practices. For these reasons, surveillance systems for HAI, including AMR patterns, are an essential component of both national and facility IPC programmes. National IPC surveillance systems also feed in to general public health capacity building and the strengthening of essential public health functions. However, a recent WHO survey on the global situational analysis of AMR, showed that many regions reported poor laboratory capacity, infrastructure, and data management as impediments to surveillance [16]. In our inventory of IPC national strategy or action plan documents, most (79%) contained guidance relating to the establishment of priorities for surveillance, despite some regional variations. Of note, only 52% of documents addressed the need for standardized definitions with clear gaps in recommending surveillance in the context of outbreak response and detection.

### Acute health care facility level

#### Recommendation

The panel recommends that facility-based HAI surveillance should be performed to guide IPC interventions and detect outbreaks, including AMR surveillance, with timely feedback of results to health care workers and stakeholders and through national networks.

***(Strong recommendation, very low quality of evidence)***

Evaluation of the evidence from 13 studies (11 non-controlled before-after [39–49], one interrupted time series [50] and one qualitative study [51]) showed that a hospital-based surveillance system, especially when linked to national surveillance networks, is associated with a decrease in overall HAI, central line-associated bloodstream infections, ventilator-associated pneumonia, surgical site infection, and catheter-related urinary tract infections. The studies also emphasized that the timely feedback of results is influential in the implementation of effective IPC actions. Active surveillance with public feedback as part of a methicillin-resistant *Staphylococcus aureus* (MRSA) care bundle strategy was associated with a decrease in MRSA infections in a hospital in Singapore [50]. One qualitative study explored the importance of surveillance and feedback to stakeholders and found that they were very influential in the implementation of an IPC programme targeting ventilator-associated pneumonia [51]. All studies were from high-income countries. The overall quality of evidence was very low given the study designs and the high risk of bias. However, given the importance of surveillance not only for reducing HAI and the early detection of outbreaks, but also for awareness-raising about the importance of HAI and AMR, the panel decided to strongly recommend that HAI surveillance with timely feedback of results should be performed in acute health care facilities to guide IPC interventions.

### National level

#### Recommendation

The panel recommends that national HAI surveillance programmes and networks that include mechanisms for timely data feedback and with the potential to be used for benchmarking purposes should be established to reduce HAI and AMR.

***(Strong recommendation, very low quality of evidence)***

Evaluation of the evidence from one trial (randomized controlled study [52]) shows that when HAI surveillance programmes introduce mechanisms for timely feedback and national benchmarking in the context of a sub-national network, there is a significant reduction in HAI rates. Although they did not meet the EPOC quality criteria, a number of additional articles clearly showed the benefits of national surveillance and feedback to reduce HAIs. Given the importance of surveillance per se to reduce HAIs and to guide effective IPC interventions, the panel decided to strongly recommend that national HAI surveillance programmes including mechanisms for timely feedback should be established to reduce HAI and AMR and be used for benchmarking purposes, despite the limited evidence available. However, the panel recognized that their implementation is resource-intensive (both financial and human resources), particularly in LMICs.

## Core component 5: Multimodal strategies

Over the past decade, studies in IPC and implementation research have demonstrated that best practice interventions are most effective when applying several interventions/approaches integrated in a multimodal strategy. At its core, a multimodal implementation strategy supports the translation of evidence and guideline recommendations into practice within health care with a view to changing health care worker behaviour.

A multimodal strategy consists of several elements or components (three or more - usually five) implemented in an integrated manner. It includes tools, such as bundles and checklists, developed by multidisciplinary teams that take into account local conditions. The five most common components include: (i) system change (improving equipment availability and infrastructure at the point of care) to facilitate best practice; (ii) education and training of health care workers and key stakeholders (e.g. managers and hospital administrators); (iii) monitoring of practices, processes, and outcomes and providing timely feedback; (iv) improved communication (e.g. reminders in the workplace or videos); and (v) culture change by fostering a safety climate [53]. It is widely accepted that focusing on one approach (component) only will not achieve or sustain behaviour change. A national approach in support of the implementation of multimodal IPC improvement efforts is recognized as having key benefits compared to localized efforts alone. For the purposes of this work, “national” was considered to embrace both national and/or subnational (e.g. state-wide) activity.

### Acute health care facility level

#### Recommendation

The panel recommends implementing IPC activities using multimodal strategies to improve practices and reduce HAI and AMR.

***(Strong recommendation, low quality of evidence)***

Evaluation of the evidence from 44 studies (13 non-controlled before-after [22, 37, 54–64], eight non-controlled cohort trials [65–72], ten interrupted time series [18, 25, 27, 29, 50, 73–77], four qualitative [31, 78–80], three randomized controlled trials [81–83], two controlled before-after [35, 84], two mixed methods [38, 85], one non-controlled interrupted time series [86] and one stepped wedge [87]) showed that implementing IPC activities at facility level using multimodal strategies is effective to improve IPC practices and reduce HAI. This was particularly relevant for hand hygiene compliance, central line-associated bloodstream infection, ventilator-associated pneumonia and infections caused by MRSA and *Clostridium difficile*. Multimodal strategies included the following components: system change; education; awareness raising; bundle-based strategies; promotion of a patient safety culture, including leadership engagement, identification of champions and positive reinforcement strategies; and increased accountability via monitoring and timely feedback. Forty studies were from high-income countries [18, 25, 27, 31, 37, 38, 50, 54–60, 62–87], two from one upper-middle-income country [29, 35], and one from a Lower-Middle-Income Country [61].

The overall quality of evidence was low given the medium- to high-risk of bias across studies and the different study designs. Based on this evidence, the panel strongly recommended that the implementation of IPC activities should be done using multimodal strategies in an effort to improve care practices, reduce HAI, and combat AMR.

### National level

#### Recommendation

The panel recommends that national IPC programmes should coordinate and facilitate the implementation of IPC activities through multimodal strategies on a nationwide or sub-national level.

***(Strong recommendation, low quality of evidence)***

Evaluation of the evidence from 14 studies (seven interrupted time series [67, 88–93], four controlled before-after [63, 94–96], two randomized controlled trials [83, 97] and one non-randomized controlled trials [98]) shows that the national roll-out of multimodal strategies is associated with reductions in central line-associated bloodstream infection, MRSA infections, and increased hand hygiene compliance. By contrast, no significant difference in surgical site infections rates was observed. The elements within the national multimodal strategies varied, but they were evaluated as a collective whole. The number of elements ranged from two to eight. The most frequently cited elements were the implementation of a care bundle with the provision of training and campaign materials to support the implementation [63, 67, 83, 88, 89, 94–98]. All studies were from high-income countries. The overall quality of evidence was low given the medium- to high-risk of bias across studies.

Given the relatively good number of national studies identified and the conviction that multimodal strategies are an innovative and effective approach not only to reduce HAIs, but also to achieve broader patient safety improvement, the panel decided to strongly recommend that IPC activities should be implemented under the coordination and facilitation of the national IPC programme using multimodal strategies in an effort to improve care practices and reduce HAI and combat AMR.

## Core component 6: Monitoring/audit of IPC practices and feedback

IPC interventions require the consistent practice of preventive procedures, such as hand hygiene, respiratory hygiene, use of surgical antimicrobial prophylaxis, the aseptic manipulation of invasive devices, and many others. The appropriateness with which these procedures are performed depends on the individual health care worker’s behaviour and the availability of the appropriate resources and infrastructures. To identify deviations from requirements and to improve performance and compliance, the frequent assessment of working practices is necessary by using standardized auditing, indicator monitoring, and feedback.

The monitoring and evaluation of national programmes is important to track the effectiveness of national policies and strategies, including providing critical information to support implementation and future development and improvement. Our inventory showed that 72% of national IPC documents across all WHO regions addressed the need for both national and facility level monitoring and evaluation. These ranged from 56% in the Western Pacific Region to 86% in the South-East Asia Region. Therefore, national monitoring and evaluation is currently being recognized as a means to determine the effectiveness of IPC programmes.

### Acute health care facility level

#### Recommendation

The panel recommends that regular monitoring/audit and timely feedback of health care practices according to IPC standards should be performed to prevent and control HAIs and AMR at the facility level. Feedback should be provided to all audited persons and relevant staff.

***(Strong recommendation, low quality of evidence)***

Evaluation of the evidence from six studies (one randomized controlled trial [99], two controlled before-after [100, 101], one interrupted time series [50], and two non-controlled before-after [102, 103]) showed that the regular monitoring/auditing of IPC practices paired with regular feedback (individually and/or team/unit) is effective to increase adherence to care practices and to decrease overall HAI. Five studies were from high-income countries [50, 99, 101–103] and one from an upper-middle-income country [100]. Due to varied methodologies and different outcomes measured, no meta-analysis was performed. The overall quality of evidence was low given the medium- to high-risk of bias across studies and the different study designs. However, the importance of the monitoring and feedback of IPC practices to demonstrate existing gaps and achieve health care workers’ behavioural change toward good practices was recognized. Therefore, the panel strongly recommended that audits and timely feedback to staff who influence the change of health care practices according to IPC standards should be performed regularly for the prevention of HAI and AMR.

### National level

#### Recommendation

The panel recommends that a national IPC monitoring and evaluation programme should be established to assess the extent to which standards are being met and activities are being performed according to the programme’s goals and objectives. Hand hygiene monitoring with feedback should be considered as a key performance indicator at the national level.

***(Strong recommendation, moderate quality of evidence)***

Evaluation of the evidence from one sub-national study (randomized controlled trial [81]) showed that the national feedback of IPC monitoring data is effective to increase adherence to best practice in individual facilities and to decrease the device-associated infection rate. The quality of this study was graded as moderate. Despite the limited evidence, the panel agreed that monitoring and evaluation should be an activity driven and coordinated by the national IPC programme and that this would be a strong recommendation. The panel also proposed that hand hygiene be considered as a key indicator for all national IPC programmes.

## Core component 7: Workload, staffing and bed occupancy

Overcrowding in health care facilities is recognized as being a public health issue that is associated with disease transmission. A combination of factors should be considered when determining the patient-to-bed ratio and the health care worker-to-patient ratio, including patient acuity, health care demand, and the availability of a trained workforce. These factors may interfere with providing optimal staff-to-patient ratio, which could potentially lead to increased rates of HAI and the spread of AMR.

### Acute health care facility level only

#### Recommendation

The panel recommends that the following elements should be adhered to in order to reduce the risk of HAI and the spread of AMR: (1) bed occupancy should not exceed the standard capacity of the facility; (2) health care worker staffing levels should be adequately assigned according to patient workload.

***(Strong recommendation, very low quality of evidence)***

Evaluation of the evidence from 19 studies (12 non-controlled cohort [104–115], three case-control studies [116–118], one interrupted time series [119], one non-controlled interrupted time series [120], one mixed methods [121] and one cross-sectional [122]) showed that bed occupancy exceeding the standard capacity of the facility is associated with the increased risk of HAI in acute care facilities, in addition to inadequate health care worker staffing levels. Studies were all from high-income countries. MRSA transmission and infection were associated with bed occupancy in six studies [106–109, 119, 123] and the nurse-to-patient ratio in seven studies [105, 112, 115–117, 120, 121]. Three studies reported that increases in nurse-to-patient ratios resulted in reduced HAI [110, 111, 113], while inadequate adherence to hand hygiene protocols was associated with low staffing levels in one study and with high workload in another [114, 122]. The overall quality of the evidence was very low. However, the panel unanimously decided to strongly recommend adherence to bed occupancy not exceeding the standard capacity of the facility and adequate health care worker staffing levels according to patient workload. When elaborating this recommendation, the panel considered the importance of these topics not only for reducing the risk of HAI and the spread of AMR, but also for achieving quality health service delivery in the context of universal health coverage.

## Core component 8. Built environment, materials and equipment for IPC at the facility level

Safe effective performance in the delivery of day-to-day patient care and treatment is crucial for optimal outcomes, both for patients and health care workers’ health and safety. In an effort to promote effective and standardized clinical practice in accordance with guidelines, emphasis should be placed on optimizing the health care environment to ensure a work system that supports the effective implementation of IPC practices.

Hand hygiene is considered as the cornerstone of clinical practice and an essential measure for the prevention of HAI and the spread of AMR. WHO issued global guidelines including evidence- and consensus-based recommendations on hand hygiene in health care [54], together with an implementation strategy and toolkit (http://www.who.int/gpsc/5may/tools/en/). These are considered to be the gold standard and are implemented in many countries worldwide. A multimodal strategy is the internationally accepted approach to achieve hand hygiene behavioural change (component 5). One of the five elements of the WHO hand hygiene improvement strategy relates to the work system within which hand hygiene takes place, i.e. an environment including an infrastructure and materials that facilitate compliance at the point of care.

### Acute health care facility level only

#### Good practice statement

General principle - patient care activities should be undertaken in a clean and/or hygienic environment that facilitates practices related to the prevention and control of HAI, as well as AMR, including all elements around the WASH infrastructure and services and the availability of appropriate IPC materials and equipment.

Ensuring the provision of adequate appropriate materials, items and equipment in relation to WASH services and their optimal placement or position are recognised as critical elements of human factors engineering (ergonomics), which support their appropriate use and increases compliance with good practices. Ultimately, this contributes to the effective implementation and the attainment of the desired behaviour to support IPC.

Several environmental issues are of concern for IPC. The most relevant are those that deal with some features of the building design and WASH-related conditions in the health care facility. The panel deemed it essential to describe the appropriate water and sanitation services, environment, and materials and equipment for IPC as a core component of effective IPC programmes in health care facilities. Therefore, despite the absence of specific studies testing the effectiveness of these important aspects as interventions to reduce HAI and AMR, the panel decided to formulate a good practice statement to outline the most relevant elements for a safe environment supporting appropriate IPC practices.

Conversely, specific evidence was available on the importance of hand hygiene facilities. Therefore, the panel also decided to develop a specific recommendation related to hand hygiene facilities.

#### Recommendation

The panel recommends that materials and equipment to perform appropriate hand hygiene should be readily available at the point of care.

***(Strong recommendation, very low quality of evidence)***

Evaluation of the evidence from 11 studies (one randomized controlled trial [124], four non-controlled before-after [62, 125–127],and one qualitative study [80]) showed that the ready availability of equipment and products at the point of care leads to an increase in compliance with good practices and the reduction of HAI. In six of the 11 studies, the intervention consisted of the ready availability and optimal placement of hand hygiene materials and equipment in areas designated for patient care or where other health care procedures are performed and led to a significant increase in hand hygiene compliance. All studies were performed in high-income countries only. The overall quality of evidence was very low, but the panel decided to recommend that materials and equipment to perform hand hygiene should be readily available at all points of care.

## Conclusions

We discussed the evidence for an interrelated set of measures identified by an expert panel as contributing to reducing the risk of HAI and combating AMR at the national and acute health care facility level. It is important to note that although the recommendations for the facility level focus on acute health care facilities, the core principles and practices of IPC as a countermeasure to the development of HAI are common to any facility where health care is delivered. Therefore, these guidelines should be considered with some adaptations by community, primary care and long-term care facilities as they develop and review their IPC programmes. Furthermore, while legal, policy and regulatory contexts may vary, these guidelines are relevant to both high- and low-resource settings as the need for effective IPC programmes is universal across different cultures and contexts.

Indeed, adaptation to the local context, taking into account available resources, culture and public health needs, will be important in the implementation of the guideline recommendations. There is also a particular need for careful evaluation of feasibility and costs in low-resource settings. Adoption should be facilitated by sound implementation strategies and practical tools. It is important to note that WHO is about to develop an implementation strategy and tools for the IPC core components at the national and facility level, including specific guidance for settings with limited resources.

## Additional files

Additional file 1: Appendix 1. Search terms of the systematic review and evidence-based guidance on the organization of hospital infection control programmes (SIGHT) and its update. (DOCX 884 kb)
Additional file 2: Appendix 2. Search terms of the systematic literature review on core components for infection prevention and control programmes at the national level. (DOCX 1010 kb)

## Abbreviations

AMR: Antimicrobial resistance
HAI: Health care-associated infection
IPC: Infection prevention and control
LMIC: Low- and middle-income countries
SIGHT: Systematic review and evidence-based guidance on organization of hospital infection control programmes
WASH: Water, sanitation and hygiene
WHO: World Health Organization

## References

[1] Report on the endemic burden of healthcare-associated infection worldwide. Geneva: World Health Organization; 2011. http://apps.who.int/iris/bitstream/10665/80135/1/9789241501507_eng.pdf. Accessed 13 Oct 2016.

[2] Allegranzi B, Bagheri Nejad S, Combescure C, Graafmans W, Attar H, Donaldson L (2011). Burden of endemic health-care-associated infection in developing countries: systematic review and meta-analysis. Lancet.

[3] The direct medical costs of healthcare-associated infections in U.S. hospitals and the benefits of prevention. Atlanta (GE): Center for Disease Control and Prevention; 2009. https://www.cdc.gov/HAI/pdfs/hai/Scott_CostPaper.pdf. Accessed 13 Oct 2016.

[4] Annual epidemiological report on communicable diseases in Europe 2008. Report on the state of communicable diseases in the EU and EEA/EFTA countries. Stockholm: European Centre for Disease Prevention and Control; 2008. http://ecdc.europa.eu/en/publications/Publications/0812_SUR_Annual_Epidemiological_Report_2008.pdf. Accessed 13 Oct 2016.

[5] The evolving threat of antimicrobial resistance - options for action. Geneva: World Health Organization; 2012. http://www.who.int/patientsafety/implementation/amr/en/. Accessed 13 Oct 2016.

[6] UK Five year antimicrobial resistance strategy 2013 to 2018. London: Department of Health; 2013. https://www.gov.uk/government/uploads/system/uploads/attachment_data/file/244058/20130902_UK_5_year_AMR_strategy.pdf. Accessed 2 Nov 2016.

[7] Report on the consultative meeting on antimicrobial resistance for countries in the Eastern Mediterranean Region: from policies to action. Geneva: World Health Organization; 2013. http://applications.emro.who.int/docs/IC_Meet_Rep_2014_EN_15210.pdf. Accessed 2 Nov 2016.

[8] International Health Regulations (2005). 2nd ed.

[9] Global action plan on antimicrobial resistance. Geneva: World Health Organization; 2015. http://www.wpro.who.int/entity/drug_resistance/resources/global_action_plan_eng.pdf. Accessed 13 Oct 2016.

[10] Core components for infection prevention and control programmes. Geneva: World Health Organization; 2009. http://www.who.int/csr/resources/publications/WHO_HSE_EPR_2009_1/en/index.html. Accessed 13 Oct 2016. 27977095

[11] Zingg W, Holmes A, Dettenkofer M, Goetting T, Secci F, Clack L (2015). Hospital organisation, management, and structure for prevention of health-care-associated infection: a systematic review and expert consensus. Lancet Infect Dis.

[12] WHO handbook for guideline development. 2nd ed. Geneva: World Health Organization; 2014. http://apps.who.int/iris/bitstream/10665/75146/1/9789241548441_eng.pdf. Accessed 13 Oct 2016.

[13] Zingg W, Castro-Sanchez E, Secci FV, Edwards R, Drumright LN, Sevdalis N (2016). Innovative tools for quality assessment: integrated quality criteria for review of multiple study designs (ICROMS). Public Health.

[14] Effective Practice and Organisation of Care (EPOC) (2015). Suggested risk of bias criteria for EPOC reviews. EPOC Resources for review authors.

[15] Guyatt GH, Schunemann HJ, Djulbegovic B, Akl EA (2015). Guideline panels should not GRADE good practice statements. J Clin Epidemiol.

[16] Worldwide country situation analysis: response to antimicrobial resistance. Geneva: World Health Organization; 2015. http://apps.who.int/iris/bitstream/10665/163468/1/9789241564946_eng.pdf?ua=1&ua=1. Accessed 13 Oct 2016.

[17] Haley RW, Culver DH, White JW, Morgan WM, Emori TG, Munn VP (1985). The efficacy of infection surveillance and control programs in preventing nosocomial infections in US hospitals. Am J Epidemiol.

[18] Mermel LA, Jefferson J, Blanchard K, Parenteau S, Mathis B, Chapin K (2013). Reducing *Clostridium difficile* incidence, colectomies, and mortality in the hospital setting: a successful multidisciplinary approach. Jt Comm J Qual Patient Saf.

[19] Larson EL, Quiros D, Lin SX (2007). Dissemination of the CDC’s hand hygiene guideline and impact on infection rates. Am J Infect Control.

[20] Rosenthal VD, Guzman S, Safdar N (2004). Effect of education and performance feedback on rates of catheter-associated urinary tract infection in intensive care units in Argentina. Infect Control Hosp Epidemiol.

[21] Rosenthal VD, McCormick RD, Guzman S, Villamayor C, Orellano PW (2003). Effect of education and performance feedback on handwashing: the benefit of administrative support in Argentinean hospitals. Am J Infect Control.

[22] Rosenthal VD, Guzman S, Safdar N (2005). Reduction in nosocomial infection with improved hand hygiene in intensive care units of a tertiary care hospital in Argentina. Am J Infect Control.

[23] Rubinson L, Wu AW, Haponik EE, Diette GB (2005). Why is it that internists do not follow guidelines for preventing intravascular catheter infections?. Infect Control Hosp Epidemiol.

[24] Quiros D, Lin S, Larson EL (2007). Attitudes toward practice guidelines among intensive care unit personnel: a cross-sectional anonymous survey. Heart Lung.

[25] Allen GB, Miller V, Nicholas C, Hess S, Cordes MK, Fortune JB (2014). A multitiered strategy of simulation training, kit consolidation, and electronic documentation is associated with a reduction in central line-associated bloodstream infections. Am J Infect Control.

[26] Gerolemou L, Fidellaga A, Rose K, Cooper S, Venturanza M, Aqeel A (2014). Simulation-based training for nurses in sterile techniques during central vein catheterization. Am J Crit Care.

[27] Johnson L, Grueber S, Schlotzhauer C, Phillips E, Bullock P, Basnett J (2014). A multifactorial action plan improves hand hygiene adherence and significantly reduces central line-associated bloodstream infections. Am J Infect Control.

[28] Kwok YL, Callard M, McLaws ML (2015). An automated hand hygiene training system improves hand hygiene technique but not compliance. Am J Infect Control.

[29] Viana WN, Bragazzi C, Couto de Castro JE, Alves MB, Rocco JR (2013). Ventilator-associated pneumonia prevention by education and two combined bedside strategies. ISQua.

[30] Joshi SC, Diwan V, Tamhankar AJ, Joshi R, Shah H, Sharma M (2012). Qualitative study on perceptions of hand hygiene among hospital staff in a rural teaching hospital in India. J Hosp Infect.

[31] Nicol PW, Watkins RE, Donovan RJ, Wynaden D, Cadwallader H (2009). The power of vivid experience in hand hygiene compliance. J Hosp Infect.

[32] Sladek RM, Bond MJ, Phillips PA (2008). Why don’t doctors wash their hands? A correlational study of thinking styles and hand hygiene. Am J Infect Control.

[33] Turnberg W, Daniell W, Simpson T, Van Buren J, Seixas N, Lipkin E (2009). Personal healthcare worker (HCW) and work-site characteristics that affect HCWs’ use of respiratory-infection control measures in ambulatory healthcare settings. Infect Control Hosp Epidemiol.

[34] Barsuk JH, Cohen ER, Feinglass J, McGaghie WC, Wayne DB (2009). Use of simulation-based education to reduce catheter-related bloodstream infections. Arch Intern Med.

[35] Marra AR, Guastelli LR, de Araujo CM, dos Santos JL, Lamblet LC, Silva M (2010). Positive deviance: a new strategy for improving hand hygiene compliance. Infect Control Hosp Epidemiol.

[36] Sherertz RJ, Ely EW, Westbrook DM, Gledhill KS, Streed SA, Kiger B (2000). Education of physicians-in-training can decrease the risk for vascular catheter infection. Ann Intern Med.

[37] Zingg W, Imhof A, Maggiorini M, Stocker R, Keller E, Ruef C (2009). Impact of a prevention strategy targeting hand hygiene and catheter care on the incidence of catheter-related bloodstream infections. Crit Care Med.

[38] Thomas M, Gillespie W, Krauss J, Harrison S, Medeiros R, Hawkins M (2005). Focus group data as a tool in assessing effectiveness of a hand hygiene campaign. Am J Infect Control.

[39] Barwolff S, Sohr D, Geffers C, Brandt C, Vonberg RP, Halle H (2006). Reduction of surgical site infections after caesarean delivery using surveillance. J Hosp Infect.

[40] Brandt C, Sohr D, Behnke M, Daschner F, Ruden H, Gastmeier P (2006). Reduction of surgical site infection rates associated with active surveillance. Infect Control Hosp Epidemiol.

[41] Gastmeier P, Behnke M, Schwab F, Geffers C (2011). Benchmarking of urinary tract infection rates: experiences from the intensive care unit component of the German national nosocomial infections surveillance system. J Hosp Infect.

[42] Gastmeier P, Geffers C, Brandt C, Zuschneid I, Sohr D, Schwab F (2006). Effectiveness of a nationwide nosocomial infection surveillance system for reducing nosocomial infections. J Hosp Infect.

[43] Gastmeier P, Schwab F, Sohr D, Behnke M, Geffers C (2009). Reproducibility of the surveillance effect to decrease nosocomial infection rates. Infect Control Hosp Epidemiol.

[44] Gastmeier P, Sohr D, Brandt C, Eckmanns T, Behnke M, Ruden H (2005). Reduction of orthopaedic wound infections in 21 hospitals. Arch Orthop Trauma Surg.

[45] Geubbels EL, Nagelkerke NJ, Mintjes-De Groot AJ, Vandenbroucke-Grauls CM, Grobbee DE, De Boer AS (2006). Reduced risk of surgical site infections through surveillance in a network. Int J Qual Health Care.

[46] L’Heriteau F, Olivier M, Maugat S, Joly C, Merrer J, Thaler F (2007). Impact of a five-year surveillance of central venous catheter infections in the REACAT intensive care unit network in France. J Hosp Infect.

[47] Rosenthal VD, Guzman S, Pezzotto SM, Crnich CJ (2003). Effect of an infection control program using education and performance feedback on rates of intravascular device-associated bloodstream infections in intensive care units in Argentina. Am J Infect Control.

[48] Schwab F, Geffers C, Barwolff S, Ruden H, Gastmeier P (2007). Reducing neonatal nosocomial bloodstream infections through participation in a national surveillance system. J Hosp Infect.

[49] Zuschneid I, Schwab F, Geffers C, Behnke M, Ruden H, Gastmeier P (2007). Trends in ventilator-associated pneumonia rates within the German nosocomial infection surveillance system (KISS). Infect Control Hosp Epidemiol.

[50] Fisher D, Tambyah PA, Lin RT, Jureen R, Cook AR, Lim A (2013). Sustained meticillin-resistant *Staphylococcus aureus* control in a hyper-endemic tertiary acute care hospital with infrastructure challenges in Singapore. J Hosp Infect.

[51] Pinto A, Burnett S, Benn J, Brett S, Parand A, Iskander S (2011). Improving reliability of clinical care practices for ventilated patients in the context of a patient safety improvement initiative. J Eval Clin Pract.

[52] McKinley LL, Moriarty HJ, Short TH, Johnson CC (2003). Effect of comparative data feedback on intensive care unit infection rates in a Veterans Administration Hospital Network System. Am J Infect Control.

[53] WHO guidelines on hand hygiene in health care. Geneva: World Health Organization; 2009. http://apps.who.int/iris/bitstream/10665/44102/1/9789241597906_eng.pdf. Accessed 13 Oct 2016.

[54] Bouadma L, Deslandes E, Lolom I, Le Corre B, Mourvillier B, Regnier B (2010). Long-term impact of a multifaceted prevention program on ventilator-associated pneumonia in a medical intensive care unit. Clin Infect Dis.

[55] Brown SM, Lubimova AV, Khrustalyeva NM, Shulaeva SV, Tekhova I, Zueva LP (2003). Use of an alcohol-based hand rub and quality improvement interventions to improve hand hygiene in a Russian neonatal intensive care unit. Infect Control Hosp Epidemiol.

[56] Costers M, Viseur N, Catry B, Simon A. Four multifaceted countrywide campaigns to promote hand hygiene in Belgian hospitals between 2005 and 2011: impact on compliance to hand hygiene. Euro Surveill. 2012;17:1–6. 10.2807/ese.17.18.20161-en 22587957

[57] Doron SI, Kifuji K, Hynes BT, Dunlop D, Lemon T, Hansjosten K (2011). A multifaceted approach to education, observation, and feedback in a successful hand hygiene campaign. Jt Comm J Qual Patient Saf.

[58] Henderson DM, Staiger TO, Peterson GN, Sinanan MN, Angiulo CL, Makarewicz VA (2012). A collaborative, systems-level approach to eliminating healthcare-associated MRSA, central-line-associated bloodstream infections, ventilator-associated pneumonia, and respiratory virus infections. J Healthc Qual.

[59] Koll BS, Straub TA, Jalon HS, Block R, Heller KS, Ruiz RE (2008). The CLABs collaborative: a regionwide effort to improve the quality of care in hospitals. Jt Comm J Qual Patient Saf.

[60] Lederer JW, Best D, Hendrix V (2009). A comprehensive hand hygiene approach to reducing MRSA health care-associated infections. Jt Comm J Qual Patient Saf.

[61] Mathai AS, George SE, Abraham J (2011). Efficacy of a multimodal intervention strategy in improving hand hygiene compliance in a tertiary level intensive care unit. Indian J Crit Care Med.

[62] McLaws ML, Pantle AC, Fitzpatrick KR, Hughes CF (2009). Improvements in hand hygiene across New South Wales public hospitals: clean hands save lives, part III. Med J Aust.

[63] McLaws ML, Pantle AC, Fitzpatrick KR, Hughes CF (2009). More than hand hygiene is needed to affect methicillin-resistant *Staphylococcus aureus* clinical indicator rates: clean hands save lives, part IV. Med J Aust.

[64] Pittet D, Hugonnet S, Harbarth S, Mourouga P, Sauvan V, Touveneau S (2000). Effectiveness of a hospital-wide programme to improve compliance with hand hygiene. Lancet.

[65] Eggimann P, Harbarth S, Constantin MN, Touveneau S, Chevrolet JC, Pittet D (2000). Impact of a prevention strategy targeted at vascular-access care on incidence of infections acquired in intensive care. Lancet.

[66] Grayson ML, Russo PL, Cruickshank M, Bear JL, Gee CA, Hughes CF (2011). Outcomes from the first 2 years of the Australian National Hand Hygiene Initiative. Med J Aust.

[67] Jain R, Kralovic SM, Evans ME, Ambrose M, Simbartl LA, Obrosky DS (2011). Veterans Affairs initiative to prevent methicillin-resistant Staphylococcus aureus infections. N Engl J Med.

[68] Jamal A, O’Grady G, Harnett E, Dalton D, Andresen D (2012). Improving hand hygiene in a paediatric hospital: a multimodal quality improvement approach. BMJ Qual Saf.

[69] Kirkland KB, Homa KA, Lasky RA, Ptak JA, Taylor EA, Splaine ME (2012). Impact of a hospital-wide hand hygiene initiative on healthcare-associated infections: results of an interrupted time series. BMJ Qual Saf.

[70] Peredo R, Sabatier C, Villagra A, Gonzalez J, Hernandez C, Perez F (2010). Reduction in catheter-related bloodstream infections in critically ill patients through a multiple system intervention. Eur J Clin Microbiol Infect Dis.

[71] Pontivivo G, Rivas K, Gallard J, Yu N, Perry L (2012). A new approach to improving hand hygiene practice in an inner city acute hospital in Australia. Healthcare Infect.

[72] Render ML, Hasselbeck R, Freyberg RW, Hofer TP, Sales AE, Almenoff PL (2011). Reduction of central line infections in Veterans Administration intensive care units: an observational cohort using a central infrastructure to support learning and improvement. BMJ Qual Saf.

[73] Al-Tawfiq JA, Abed MS, Al-Yami N, Birrer RB (2013). Promoting and sustaining a hospital-wide, multifaceted hand hygiene program resulted in significant reduction in health care-associated infections. Am J Infect Control.

[74] Higgins A, Hannan MM (2013). Improved hand hygiene technique and compliance in healthcare workers using gaming technology. J Hosp Infect.

[75] Shepherd EG, Kelly TJ, Vinsel JA, Cunningham DJ, Keels E, Beauseau W (2015). Significant reduction of central-line associated bloodstream infections in a network of diverse neonatal nurseries. J Pediatr.

[76] Talbot TR, Johnson JG, Fergus C, Domenico JH, Schaffner W, Daniels TL (2013). Sustained improvement in hand hygiene adherence: utilizing shared accountability and financial incentives. Infect Control Hosp Epidemiol.

[77] Mayer J, Mooney B, Gundlapalli A, Harbarth S, Stoddard GJ, Rubin MA (2011). Dissemination and sustainability of a hospital-wide hand hygiene program emphasizing positive reinforcement. Infect Control Hosp Epidemiol.

[78] Creamer E (2000). Examining the care of patients with peripheral venous cannulas. Br J Nurs.

[79] Damschroder LJ, Banaszak-Holl J, Kowalski CP, Forman J, Saint S, Krein SL (2009). The role of the champion in infection prevention: results from a multisite qualitative study. Qual Saf Health Care.

[80] Jang JH, Wu S, Kirzner D, Moore C, Youssef G, Tong A (2010). Focus group study of hand hygiene practice among healthcare workers in a teaching hospital in Toronto, Canada. Infect Control Hosp Epidemiol.

[81] Fuller C, Michie S, Savage J, McAteer J, Besser S, Charlett A (2012). The Feedback Intervention Trial (FIT)--improving hand-hygiene compliance in UK healthcare workers: a stepped wedge cluster randomised controlled trial. PLoS One.

[82] Huis A, Schoonhoven L, Grol R, Donders R, Hulscher M, Achterberg TV. Impact of a team and leaders-directed strategy to improve nurses’ adherence to hand hygiene guidelines: a cluster randomised trial. Int J Nrs Stud. 2013;50:464–474. 10.1016/j.ijnurstu.2012.08.004 22939048

[83] Stevenson KB, Searle K, Curry G, Boyce JM, Harbarth S, Stoddard GJ (2014). Infection control interventions in small rural hospitals with limited resources: results of a cluster-randomized feasibility trial. Antimicrob Resist Infect Contr.

[84] Lieber SR, Mantengoli E, Saint S, Fowler KE, Fumagalli C, Bartolozzi D (2014). The effect of leadership on hand hygiene: assessing hand hygiene adherence prior to patient contact in 2 infectious disease units in Tuscany. Infect Control Hosp Epidemiol.

[85] Creedon SA (2006). Health care workers’ hand decontamination practices: an Irish study. Clin Nurs Res.

[86] DePalo VA, McNicoll L, Cornell M, Rocha JM, Adams L, Pronovost PJ (2010). The Rhode Island ICU collaborative: a model for reducing central line-associated bloodstream infection and ventilator-associated pneumonia statewide. Qual Saf Health Care.

[87] Rodriguez V, Giuffre C, Villa S, Almada G, Prasopa-Plaizier N, Gogna M (2015). A multimodal intervention to improve hand hygiene in ICUs in Buenos Aires, Argentina: A stepped wedge trial. Int J Qual Health Care.

[88] Hansen S, Schwab F, Schneider S, Sohr D, Gastmeier P, Geffers C (2014). Time-series analysis to observe the impact of a centrally organized educational intervention on the prevention of central-line-associated bloodstream infections in 32 German intensive care units. J Hosp Infect.

[89] Newitt S, Myles PR, Birkin JA, Maskell V, Slack RCB, Nguyen-Van-Tam JS (2015). Impact of infection control interventions on rates of *Staphylococcus aureus* bacteraemia in National Health Service acute hospitals, East Midlands, UK, using interrupted time-series analysis. J Hosp Infect.

[90] Miller MR, Griswold M, Harris JM, Yenokyan G, Huskins WC, Moss M (2010). Decreasing PICU catheter-associated bloodstream infections: NACHRI’s quality transformation efforts. Pediatrics.

[91] Miller MR, Niedner MF, Huskins WC, Colantuoni E, Yenokyan G, Moss M (2011). Reducing PICU central line-associated bloodstream infections: 3-year results. Pediatrics.

[92] Bundy DG, Gaur AH, Billett AL, He B, Colantuoni EA, Miller MR (2014). Preventing CLABSIs among pediatric hematology/oncology inpatients: national collaborative results. Pediatrics.

[93] Schweizer ML, Chiang H-Y, Septimus E, Moody J, Braun B, Hafner J (2015). Association of a bundled intervention with surgical site infections among patients undergoing cardiac, hip, or knee surgery. JAMA.

[94] Lipitz-Snyderman A, Steinwachs D, Needham DM, Colantuoni E, Morlock LL, Pronovost PJ (2011). Impact of a statewide intensive care unit quality improvement initiative on hospital mortality and length of stay: retrospective comparative analysis. BMJ.

[95] Reames BN, Krell RW, Campbell DA, Dimick JB (2015). A checklist-based intervention to improve surgical outcomes in Michigan: evaluation of the Keystone Surgery program. JAMA Surg.

[96] Wirtschafter DD, Powers RJ, Pettit JS, Lee HC, Boscardin WJ, Ahmad Subeh M (2011). Nosocomial infection reduction in VLBW infants with a statewide quality-improvement model. Pediatrics.

[97] Marsteller JA, Sexton JB, Hsu YJ, Hsiao CJ, Holzmueller CG, Pronovost PJ (2012). A multicenter, phased, cluster-randomized controlled trial to reduce central line-associated bloodstream infections in intensive care units. Crit Care Med.

[98] Bion J, Richardson A, Hibbert P, Beer J, Abrusci T, McCutcheon M (2013). ‘Matching Michigan’: a 2-year stepped interventional programme to minimise central venous catheter-blood stream infections in intensive care units in England. BMJ Qual Saf.

[99] Charrier L, Allochis MC, Cavallo MR, Gregori D, Cavallo F, Zotti CM (2008). Integrated audit as a means to implement unit protocols: a randomized and controlled study. J Eval Clin Pract.

[100] Moongtui W, Gauthier DK, Turner JG (2000). Using peer feedback to improve handwashing and glove usage among Thai health care workers. Am J Infect Control.

[101] Yinnon AM, Wiener-Well Y, Jerassy Z, Dor M, Freund R, Mazouz B (2012). Improving implementation of infection control guidelines to reduce nosocomial infection rates: pioneering the report card. J Hosp Infect.

[102] Cocanour CS, Peninger M, Domonoske BD, Li T, Wright B, Valdivia A (2006). Decreasing ventilator-associated pneumonia in a trauma ICU. J Trauma.

[103] Kilbride HW, Wirtschafter DD, Powers RJ, Sheehan MB (2003). Implementation of evidence-based potentially better practices to decrease nosocomial infections. Pediatrics.

[104] Alonso-Echanove J, Edwards JR, Richards MJ, Brennan P, Venezia RA, Keen J (2003). Effect of nurse staffing and antimicrobial-impregnated central venous catheters on the risk for bloodstream infections in intensive care units. Infect Control Hosp Epidemiol.

[105] Blatnik J, Lesnicar G (2006). Propagation of methicillin-resistant *Staphylococcus aureus* due to the overloading of medical nurses in intensive care units. J Hosp Infect.

[106] Borg MA (2003). Bed occupancy and overcrowding as determinant factors in the incidence of MRSA infections within general ward settings. J Hosp Infect.

[107] Cunningham JB, Kernohan WG, Rush T (2006). Bed occupancy, turnover intervals and MRSA rates in English hospitals. Br J Nurs.

[108] Cunningham JB, Kernohan WG, Sowney R (2005). Bed occupancy and turnover interval as determinant factors in MRSA infections in acute settings in Northern Ireland: 1 April 2001 to 31 March 2003. J Hosp Infect.

[109] Howie AJ, Ridley SA (2008). Bed occupancy and incidence of Methicillin-resistant Staphylococcus aureus infection in an intensive care unit. Anaesthesia.

[110] Hugonnet S, Chevrolet JC, Pittet D (2007). The effect of workload on infection risk in critically ill patients. Crit Care Med.

[111] Hugonnet S, Uckay I, Pittet D (2007). Staffing level: a determinant of late-onset ventilator-associated pneumonia. Crit Care.

[112] Hugonnet S, Villaveces A, Pittet D (2007). Nurse staffing level and nosocomial infections: empirical evaluation of the case-crossover and case-time-control designs. Am J Epidemiol.

[113] Mark BA, Harless DW, Berman WF (2007). Nurse staffing and adverse events in hospitalized children. Policy Polit Nurs Pract.

[114] Nijssen S, Bonten MJ, Franklin C, Verhoef J, Hoepelman AI, Weinstein RA (2003). Relative risk of physicians and nurses to transmit pathogens in a medical intensive care unit. Arch Intern Med.

[115] Vicca AF (1999). Nursing staff workload as a determinant of methicillin-resistant Staphylococcus aureus spread in an adult intensive therapy unit. J Hosp Infect.

[116] Fridkin SK, Pear SM, Williamson TH, Galgiani JN, Jarvis WR (1996). The role of understaffing in central venous catheter-associated bloodstream infections. Infect Control Hosp Epidemiol.

[117] Petrosillo N, Gilli P, Serraino D, Dentico P, Mele A, Ragni P (2001). Prevalence of infected patients and understaffing have a role in hepatitis C virus transmission in dialysis. Am J Kidney Dis.

[118] Robert J, Fridkin SK, Blumberg HM, Anderson B, White N, Ray SM (2000). The influence of the composition of the nursing staff on primary bloodstream infection rates in a surgical intensive care unit. Infect Control Hosp Epidemiol.

[119] Borg MA, Suda D, Scicluna E (2008). Time-series analysis of the impact of bed occupancy rates on the incidence of methicillin-resistant Staphylococcus aureus infection in overcrowded general wards. Infect Control Hosp Epidemiol.

[120] Anderson JJ, Mokracek M, Lindy CN (2009). A nursing quality program driven by evidence-based practice. Nurs Clin North Am.

[121] Virtanen M, Kurvinen T, Terho K, Oksanen T, Peltonen R, Vahtera J (2009). Work hours, work stress, and collaboration among ward staff in relation to risk of hospital-associated infection among patients. Med Care.

[122] Pittet D, Simon A, Hugonnet S, Pessoa-Silva CL, Sauvan V, Perneger TV (2004). Hand hygiene among physicians: performance, beliefs, and perceptions. Ann Intern Med.

[123] Cunningham JB, Kernohan WG, Rush T (2006). Bed occupancy, turnover interval and MRSA rates in Northern Ireland. Br J Nurs.

[124] Birnbach DJ, Nevo I, Scheinman SR, Fitzpatrick M, Shekhter I, Lombard JL (2010). Patient safety begins with proper planning: a quantitative method to improve hospital design. Qual Saf Health Care.

[125] Koff MD, Loftus RW, Burchman CC, Schwartzman JD, Read ME, Henry ES (2009). Reduction in intraoperative bacterial contamination of peripheral intravenous tubing through the use of a novel device. Anesthesiology.

[126] Thomas BW, Berg-Copas GM, Vasquez DG, Jackson BL, Wetta-Hall R (2009). Conspicuous vs customary location of hand hygiene agent dispensers on alcohol-based hand hygiene product usage in an intensive care unit. J Am Osteopath Assoc.

[127] Whitby M, McLaws ML (2004). Handwashing in healthcare workers: accessibility of sink location does not improve compliance. J Hosp Infect.

