# Supplementary material for: Core components for effective infection prevention and control programmes: new WHO evidence-based recommendations
Source: Antimicrob Resist Infect Control. 2017 Jan 10;6:6. doi: 10.1186/s13756-016-0149-9 (PMC5223492; doi:10.1186/s13756-016-0149-9)
Supplement: Additional file 1: Appendix 1. — Search terms of the systematic review and evidence-based guidance on the organization of hospital infection control programmes (SIGHT) and its update. (DOCX 884 kb) [file 13756_2016_149_MOESM1_ESM.docx]

**Appendix 1**

**Search terms of the systematic review and evidence-based guidance on the organization of hospital infection control programmes (SIGHT) and its update**

DIMENSION 1

Medline (Via OVID)

#1 cross infection.mp. or exp cross infection/

#2 infection control.mp. or exp infection nontrol/

#3 nosocomial infection.mp.

#4 healthcare-associated Infection?.mp.

#5 HAI.mp.

#6 HCAI.mp.

#7 catheter-related infection?.mp. or exp catheter-related infections/

#8 exp catheterization, central venous/

#9 CRBSI.mp.

#10 catheter-associated infection?.mp.

#11 methicillin-resistant Staphylococcus aureus.mp. or exp methicillin-resistant Staphylococcus aureus/

#12 MRSA.mp.

#13 Clostridium difficile.mp. or exp Clostridium difficile/

#14 CDI.mp.

#15 bacteremia.mp. or exp bacteremia/

#16 pneumonia, ventilator-associated.mp. or exp pneumonia, ventilator-associated/

#17 VAP.mp.

#18 handwashing.mp. or exp handwashing/

#19 exp decision making, organizational/

#20 exp efficiency, organizational/

#21 exp health facility administration/

#22 exp hospital administration/

#23 exp institutional management teams/

#24 exp management audit/

#25 exp management information systems/

#26 exp models, organizational/

#27 exp organizational culture/

#28 exp organizational innovation/

#29 exp personnel management/

#30 exp program development/

#31 exp total quality management/

#32 leadership.mp.

#33 exp infection control practitioners/

#34 exp administrative personnel/

#35 *"organization and administration"/

#36 exp infection control/or [organization & administration]

#37 exp organizational case studies/

#38 1or2or3or4or5or6or7or8or9or10or11or12or13or14or15or16or17or18

#39 exp knowledge management/

#40 exp organizational affiliation/

#41 exp career mobility/

#42 exp career mobility/

#43 exp employee discipline/

#44 exp employee grievances/

#45 exp employee incentive plans/

#46 exp employee performance appraisal/

#47 exp management quality circles/

#48 exp negotiating/

#49 exp ersonnel administration, hospital/

#50 exp personnel downsizing/ or exp personnel selection/

#51 exp personnel loyalty/

#52 exp "personnel staffing and scheduling"/

#53 exp personnel turnover/

#54 exp "salaries and fringe benefits"/

#55 exp staff development/

#56 exp workload/

#57 exp workplace/

#58 41or42or43or44or45or46or47or48or49or50or51or52or53or54or55or56 or57

#59 19or20or21or22or23or24or25or26or27or28or29or30or31or32or33or34or35or36  or 37 or 39 or 40 or 58

#60 exp hospital-physician relations/

#61 exp professional practice/

#62 risk management/

#63 safety management/

#64 59or60or61or62or63

#65 38 and 64

#66 limit 65 to (humans and yr="1996 -current" and (English or French or German or Italian or Portuguese or Spanish))

EMBASE

('organization and management'/mj OR 'hospital management'/exp OR 'organizational development'/exp OR 'organizational structure'/exp OR 'organizational efficiency'/exp OR 'leadership'/exp OR 'personnel management'/exp OR 'program development'/exp OR 'management audit' OR 'hospital administration' OR 'organizational efficiency' OR 'staff development' OR 'risk management'/exp) AND ('hospital infection'/exp OR 'cross infection'/exp OR 'infection control'/exp OR 'nosocomial infection'/exp OR 'healthcareassociated infection' OR 'hai' OR 'hcai' OR 'catheter-related infection'/exp OR 'catheter infection'/exp OR 'central venous catheterization'/exp OR 'crbsi' OR 'catheter-associated infection'/exp OR 'methicillin resistant staphylococcus aureus'/exp OR 'mrsa'/exp OR 'clostridium difficile'/exp OR 'cdi' OR 'bacteremia'/exp OR 'ventilator associated pneumonia'/exp OR 'vap' OR 'handwashing'/exp OR 'hand washing'/exp) AND ([english]/lim OR [french]/lim OR [german]/lim OR [italian]/lim OR [portuguese]/lim OR [spanish]/lim) AND [humans]/lim NOT [medline]/lim AND [embase]/lim AND [1996-2011]/py

DIMENSION 2

Medline (Via OVID)

#3 Search infection prevention [TIAB] OR infection control [TIAB]

#4 Search "infection control"[Mesh:NoExp]

#5 Search nosocomial infection* [TIAB]

#7 Search "cross Infection"[Mesh]

#8 Search (hospital acquired [TIAB] OR healthcare acquired [TIAB] OR health care acquired [TIAB] OR healthcare associated [TIAB] OR health care associated [TIAB]) AND (infection [TIAB] OR infections [TIAB])

#9 Search HAI [TIAB] OR HCAI [TIAB]

#10 Search bacteremia [TIAB] OR bacteraemia [TIAB]

#13 Search "bacteremia"[Mesh]

#14 Search (catheter associated [TIAB] OR catheter related [TIAB]) AND (infection [TIAB] OR infections  [TIAB])

#15 Search CRBSI [TIAB]

#18 Search "catheter-related infections"[Mesh] #20 Search device associated infection* [TIAB] #19 Search central line associated bloodstream infection* [TIAB] #21 Search ventilator associated pneumonia [TIAB] OR VAP [TIAB] #24 Search "pneumonia, ventilator-associated"[Mesh]

#25 Search surgical site infection* [TIAB] OR SSI [TIAB]

#27 Search "surgical wound infection"[Mesh]

#28 Search (methicillin resistant Staphylococcus aureus [TIAB] OR meticillin resistant Staphylococcus  aureus [TIAB] OR MRSA [TIAB]) AND (infection [TIAB] OR infections [TIAB])

#32 Search Clostridium difficile infection* [TIAB] OR CDI [TIAB] OR Clostridium difficile associated diarrhoea [TIAB] OR CDAD [TIAB] OR Clostridium difficile associated disease* [TIAB]

#33 Search (vancomycin resistant enterococcus [TIAB] OR vancomycin resistant enterococci [TIAB] OR  VRE [TIAB]) AND (infection [TIAB] or infections [TIAB])

#34 Search surveillance [TIAB]

#38 Search ("population surveillance"[Majr:NoExp]) OR "sentinel surveillance"[Majr]

#39 Search (#3) OR #4

#40 Search ((((((((((((((#5) OR #7) OR #8) OR #9) OR #10) OR #13) OR #14) OR #15) OR #18) OR #19)  OR #20) OR #21) OR #24) OR #25) OR #27

#41 Search ((#28) OR #32) OR #33

#42 Search ((#39) OR #40) OR #41

#43 Search (#34) OR #38

#44 Search (#42) AND #43

#45 Search (#42) AND #43 Limits: English, French, German, Italian, Spanish, Portuguese, publication date from 1996/01/01 to 2010/12/31

DIMENSION 3

Medline (via PubMed)

("education"[MeSH Terms] OR Educat$[Text Word] OR Training[Text Word]) AND ("infection control"[MeSH Terms] OR "cross infection"[MeSH Terms] OR "cross infection"[Text Word] OR "infection control"[Text Word] OR "nosocomial infection"[Text Word] OR "healthcare-Associated Infection"[Text Word] OR "healthcare-related infection"[Text Word] OR HAI[Text Word] OR HCAI[Text Word] OR "catheter-related infections"[MeSH Terms] OR "catheter-related infection?"[Text Word] OR "catheter- associated infection?"[Text Word] OR "catheterization, central venous"[MeSH Terms] OR CRBSI[Text Word] OR "methicillin-resistant Staphylococcus aureus"[MeSH Terms] OR MRSA[Text Word] OR "methicillin-resistant Staphylococcus aureus"[Text Word] OR "Clostridium difficile"[MeSH Terms] OR "Clostridium difficile"[Text Word] OR CDI[Text Word] OR "bacteremia"[MeSH Terms] OR bacteremia[Text Word] OR "pneumonia, ventilator-associated"[MeSH Terms] OR VAP[Text Word] OR "ventilator associated pneumonia"[Text Word] OR "handwashing"[MeSH Terms]OR "handwashing"[Text Word]) AND ("humans"[MeSH Terms] AND (English[lang] OR French[lang] OR German[lang] OR Italian[lang] OR Spanish[lang] OR Portuguese[lang]) AND ("1996"[PDAT] : "3000"[PDAT]))

EMBASE

('training'/exp OR 'education'/exp) AND ('hospital infection'/exp OR 'cross infection'/exp OR 'infection control'/exp OR 'nosocomial infection'/exp OR 'healthcare-associated infection' OR 'hai' OR 'hcai' OR 'catheter-related infection'/exp OR 'catheter infection'/exp OR 'central venous catheterization'/exp OR 'crbsi' OR 'catheter-associated infection'/exp OR 'methicillin resistant staphylococcus aureus'/exp OR 'mrsa'/exp OR 'clostridium difficile'/exp OR 'cdi' OR 'bacteremia'/exp OR 'ventilator associated pneumonia'/exp OR 'vap' OR 'handwashing'/exp OR 'hand washing'/exp) AND ([english]/lim OR [french]/lim OR [german]/lim OR [italian]/lim OR [portuguese]/lim OR [spanish]/lim) AND [humans]/lim AND [embase]/lim NOT [medline]/lim AND [1996- 2011]/py

DIMENSION 4

Medline (Via OVID)

#1 infection control.mp. or exp infection control/

#2 ((infection adj control) or (infection adj3 prevention) or (infection adj3 management)).mp.

#3 nosocomial infection?.mp. or exp Cross Infection/

#4 (hospital acquired infection? or healthcare associated infection? or health care  associated infection? or healthcare-associated infection? or health care-associated  infection? or HAI or HCAI).mp.

#5 methicillin resistant Staphylococcus aureus.mp. or meticillin resistant  Staphylococcus aureus.mp. or exp methicillin-resistant Staphylococcus aureus/

#6 MRSA.mp.

#7 methicillin-sensitive Staphylococcus aureus.mp. or meticillin-sensitive Staphylococcus aureus.mp.

#8 MSSA.mp.

#9 Clostridium difficile.mp. or exp Clostridium difficile/

#10 C-diff.mp. or CDI.mp. or CDAD.mp. or Clostridium difficile infection.mp. or Clostridium difficile associated disease?.mp.

#11 catheter-related infections.mp.

#12 bacter?emia.mp. or exp bacteremia/

#13 (ventilator associated pneumonia or VAP).mp.

#14 (device associated infection? or device-associated infection?).mp.

#15 surgical site infection.mp.

#16 *disease outbreaks/pc [prevention & control]

#17 handwashing/

#18 (control or prevention or management or guideline*).mp.

#19 (hand? hygiene or hand washing or isolation or screening or precaution).mp.

#20 decontamination.mp.

#21 care bundle?.mp. or bundle?.mp. or high impact intervention?.mp. or multimodal.mp.  or checklist?.mp. or care pathway?.mp.

#22 behavio?r* change.mp. or planned behavio?r*.mp.

#23 (decision making or intention? or attitude? or practic* or routine? or procedure? or  work*).mp.

#24 exp decision making/ or intention/ or exp health personnel attitudes/ or health  personnel attitude?.mp.

#25 (learning or training or education or knowledge).mp or exp education/ or exp staff development/ or professional development.mp.

#26 exp learning/ or organizational learning.mp.

#27 (workload or ((patient? adj1 staff) adj1 contact?) or practice improvement? or  professional competence? or human factor).mp.

#28 (use? adj2 medical device?).mp.

#29 motivation.mp. or exp motivation/

#30 (organizational culture or organisational culture).mp.

#31 exp organizational innovation/ or organizational innovation.mp. or organisational  innovation.mp.

#32 (organisational change or organizational change).mp.

#33 critical pathway?.mp.

#34 nurs* practice pattern?.mp.

#35 interven*.mp.

#36 (guideline? adj3 implement*).mp.

#37 exp clinical competence/

#38 clinical governance.mp. or exp clinical governance/

#39 treatment guideline?.mp. or best practice?.mp.

#40 exp guideline adherence/ or ((guideline? adj adherence) or (guideline? adj  compliance)).mp.

#41 exp clinical audit/ or audit.mp. or feedback?.mp.

#42 exp guidelines as topic/

#43 quality improvement/ or quality improvement?.mp.

#44 (service improvement or improvement methodolog*).mp.

#45 exp health personnel/ or (health personnel or healthcare professional? or healthcare  worker?).mp.

#46 (clinical staff or medical personnel or clinical personnel).mp.

#47 infection control practitioners/ or infection control practitioner?.mp. or infection  control nurse?.mp. or infection control team?.mp.

#48 exp medical staff, hospital/ or (hospital staff or hospital personnel or hospital  worker?).mp.

#49 exp nurses/ or (nurse? or nursing staff or nursing student?).mp.

#50 exp personnel, hospital/

#51 exp physicians/ or (physician? or doctor? or clinician? or surgeon? or resident? or  medical student?).mp.

#52 community healthcare.mp. or community service?.mp. or exp community health  services/ or community health care.mp. or community care.mp.

#53 exp primary health care/ or exp family practice/ or exp community health centers/ or health centre.mp. or GP practice.mp. or general practice.mp. or family practice.mp. or primary care.mp. or primary healthcare.mp. or primary health care.mp.

#54 family physicians/ or general practitioners/ or primary care physicians/ or  hospitalists/ or (general practitioner? or family practitioner? or family doctor? or  primary care doctor?.mp. or primary care physician?.mp.).mp.

#55 52or53or54

#56 3or4or5or6or7 or 8 or 9 or 10 or 11 or 12 or 13 or 14 or 15 or 16 or 17

#57 18or19or20or21

#58 56 and 57

#59 1or2or 58

#60 22or23 or 25 or 26 or 27 or 28 or 29 or 30 or 31 or 32 or 33 or 34 or 35

#61 36or37 or 38 or 40 or 41 or 42 or 43 or 44

#62 60or61

#63 45or46 or 47 or 48 or 49 or 50 or 51

#64 59and62and63

#65 64 not 55

#66 limit 65 to (yr="1996 - 2010" and (english or french or german or italian or portuguese  or spanish))

#67 remove duplicates form 66

DIMENSION 5

Medline (Via PubMed)

#1 Search "resource" OR "resources"

#5 Search ("health resources"[Mesh] OR "resource allocation"[Mesh] OR "health manpower"[Mesh]) OR "organization and administration" [Subheading]

#6 Search "requirement" OR "requirements"

#7 Search "policy" OR "policies"

#9 Search "policy making"[Mesh] OR "health policy"[Mesh] OR "economics"[Mesh]

#10 Search "strategy" OR "strategies"

#11 Search (((((#1) OR #5) OR #6) OR #7) OR #9) OR #10

#12 Search standard precaution*

#13 Search "universal precaution"

#14 Search "universal precautions"

#16 Search "universal precautions"[Mesh]

#17 Search "hand hygiene"

#18 Search hand disinfection

#19 Search "transmission based precautions"

#20 Search "transmission based" AND "precautions"

#21 Search "droplet precautions"

#22 Search contact precaution*

#24 Search airborne precaution

#25 Search airborne precautions

#26 Search "isolation precaution"

#27 Search "isolation precautions"

#29 Search "patient isolation"[Mesh]

#30 Search ((((((((((((((#12) OR #13) OR #14) OR #16) OR #17) OR #18) OR #19) OR 20) OR #21) OR  #22) OR #24) OR #25) OR #26) OR #27) OR #29

#31 Search hospital acquired infection*

#32 Search healthcare associated infection*

#33 Search nosocomial

#35 Search "cross infection"[Mesh]

#36 Search "infection control" OR "infection prevention"

#39 Search "prevention and control" [Subheading]

#40 Search Clostridium difficile

#41 Search MRSA

#42 Search VRE

#43 Search "vancomycin resistant enterococcus" OR "vancomycin resistant enterococci"

#44 Search (((((((((#31) OR #32) OR #33) OR #35) OR #36) OR #39) OR #40) OR #41) OR #42) OR #43

#45 Search (#11) AND #30

#46 Search (#45) AND #44

#47 Search (#45) AND #44 imits: English, French, German, Italian, Spanish, Portuguese, publication date from 1996/01/01 to 2010/12/31

DIMENSION 6

(“clinical audit” [MeSH Terms] OR “management audit” [MeSH Terms] OR “audit*” [Text Word] OR “auditing” [Text Word]) AND ("infection control"[MeSH Terms] OR "cross infection"[MeSH Terms] OR "cross infection"[Text Word] OR "infection control"[Text Word] OR "nosocomial infection"[Text Word] OR "healthcare-associated infection"[Text Word] OR "healthcare-related infection"[Text Word] OR HAI[Text Word] OR HCAI[Text Word] OR "catheter-related infections"[MeSH Terms] OR "catheter-related infection?"[Text Word] OR "catheter-associated infection?"[Text Word] OR "catheterization, central venous"[MeSH Terms] OR CRBSI[Text Word] OR "methicillin-resistant Staphylococcus aureus"[MeSH Terms] OR MRSA[Text Word] OR "methicillin-resistant Staphylococcus aureus"[Text Word] OR "clostridium difficile"[MeSH Terms] OR "Clostridium difficile"[Text Word] OR CDI[Text Word] OR "bacteremia"[MeSH Terms] OR bacteremia[Text Word] OR "pneumonia, ventilator-associated"[MeSH Terms] OR VAP[Text Word] OR "ventilator associated pneumonia"[Text Word] OR "handwashing"[MeSH Terms]OR "handwashing"[Text Word] OR “hand hygiene”[Text Word] OR “handrub*”[Text Word] OR “hand rub”[Text Word] OR “handrub” [Text Word])

DIMENSION 7

(“patient participation”[MeSH Terms] OR “patient empowerment”[Text Word] OR “patient empowering”[Text Word] OR ”empowering patients”[Text Word]) AND ("infection control"[MeSH Terms] OR "cross infection"[MeSH Terms] OR "cross infection"[Text Word] OR "infection control"[Text Word] OR "nosocomial infection"[Text Word] OR "healthcare-associated infection"[Text Word] OR "healthcare-related infection"[Text Word] OR HAI[Text Word] OR HCAI[Text Word] OR "catheter-related infections"[MeSH Terms] OR "catheter-related infection?"[Text Word] OR "catheter-associated infection?"[Text Word] OR "catheterization, central venous"[MeSH Terms] OR CRBSI[Text Word] OR "methicillin-resistant Staphylococcus aureus"[MeSH Terms] OR MRSA[Text Word] OR "methicillin-resistant Staphylococcus aureus"[Text Word] OR "clostridium difficile"[MeSH Terms] OR "Clostridium difficile"[Text Word] OR CDI[Text Word] OR "bacteremia"[MeSH Terms] OR bacteremia[Text Word] OR "pneumonia, ventilator-associated"[MeSH Terms] OR VAP[Text Word] OR "ventilator associated pneumonia"[Text Word] OR "handwashing"[MeSH Terms]OR "handwashing"[Text Word] OR “hand hygiene”[Text Word] OR “handrub*”[Text Word] OR “hand rub”[Text Word])

DIMENSION 8

(“target*” [Text Word] OR “target setting” [Text Word] OR “goal setting” [Text Word] OR goals [MeSH Terms] OR “organizational objectives” [MeSH Terms]) AND ("infection control"[MeSH Terms] OR "cross infection"[MeSH Terms] OR "cross infection"[Text Word] OR "infection control"[Text Word] OR "nosocomial infection"[Text Word] OR "healthcare-associated infection"[Text Word] OR "healthcare-related infection"[Text Word] OR HAI[Text Word] OR HCAI[Text Word] OR "catheter-related infections"[MeSH Terms] OR "catheter-related infection?"[Text Word] OR "catheter-associated infection?"[Text Word] OR "catheterization, central venous"[MeSH Terms] OR CRBSI[Text Word] OR "methicillin-resistant Staphylococcus aureus"[MeSH Terms] OR MRSA[Text Word] OR "methicillin-resistant Staphylococcus aureus"[Text Word] OR "clostridium difficile"[MeSH Terms] OR "Clostridium difficile"[Text Word] OR CDI[Text Word] OR "bacteremia"[MeSH Terms] OR bacteremia[Text Word] OR "pneumonia, ventilator-associated"[MeSH Terms] OR VAP[Text Word] OR "ventilator associated pneumonia"[Text Word] OR "handwashing"[MeSH Terms]OR "handwashing"[Text Word] OR “hand hygiene”[Text Word] OR “handrub*”[Text Word] OR “hand rub”[Text Word])

DIMENSION 9

‘knowledge management’/exp OR ‘knowledge management’:ti,ab OR ‘information management’/exp OR ‘knowledge’/exp OR ‘EVIDENCE BASED MEDICINE’/exp OR ‘MEDICAL RECORDS SYSTEMS, COMPUTERIZED’/exp OR ‘INFORMATION STORAGE AND RETRIEVAL’/exp OR ‘INFORMATION DISSEMINATION’/exp OR ‘USER COMPUTER INTERFACE’/exp OR ‘DIFFUSION OF INNOVATION’/exp OR ‘DECISION SUPPORT SYSTEMS, CLINICAL’/exp OR ‘MEDICAL INFORMATICS’/exp OR ‘INFORMATION SYSTEMS’/exp OR ‘ORGANIZATIONAL INNOVATION’/exp OR ‘SYSTEMS INTEGRATION’/exp OR ‘KNOWLEDGE BASES’/exp
