# Supplementary material for: Core components for effective infection prevention and control programmes: new WHO evidence-based recommendations
Source: Antimicrob Resist Infect Control. 2017 Jan 10;6:6. doi: 10.1186/s13756-016-0149-9 (PMC5223492; doi:10.1186/s13756-016-0149-9)
Supplement: Additional file 2: Appendix 2. — Search terms of the systematic literature review on core components for infection prevention and control programmes at the national level. (DOCX 1010 kb) [file 13756_2016_149_MOESM2_ESM.docx]

**Appendix 2**

**Search terms of the systematic literature review on core components for infection prevention and control programmes at the national level**

## MEDLINE (via EBSCO)

**HEALTHCARE ASSOCIATED INFECTION AND ASSOCIATED ORGANISMS**

| 1. | TI nosocomia* OR AB nosocomia* | 24,340 |
| --- | --- | --- |
| 2. | TI ("hospital acquired infection*" OR "hospital associated infection*") OR AB ("hospital acquired infection*" OR "hospital associated infection*") | 2,813 |
| 3. | TI ("healthcare acquired infection*" OR "health care acquired infection*" OR "healthcare associated infection*" OR "health care associated infection*") OR AB ("healthcare acquired infection*" OR "health care acquired infection*" OR "healthcare associated infection*" OR "health care associated infection*") | 2,541 |
| 4. | TI ( HAI OR HCAI ) OR AB ( HAI OR HCAI ) | 3,258 |
| 5. | TI "cross infection*" OR AB "cross infection*" | 2,229 |
| 6. | (MH "Cross Infection") | 48,454 |
| 7. | (MH "Infection Control") OR TI ("infection control" OR "infection prevention") OR AB ("infection control" OR "infection prevention") | 31,714 |
| 8. | (MH "Infectious Disease Transmission, Professional-to-Patient") OR (MH "Disease Transmission, Infectious") | 8,178 |
| 9. | TI ("cross transmission" OR "infectious disease transmission") OR AB ("cross transmission" OR "infectious disease transmission") | 806 |
| 10. | (MH "Catheter-Related Infections") | 2,756 |
| 11. | TI ("catheter related infection*" OR "catheter acquired infection*" OR "catheter associated infection*") OR AB ("catheter related infection*" OR "catheter acquired infection*" OR "catheter associated infection*") | 1,898 |
| 12. | TI CAUTI OR AB CAUTI | 301 |
| 13. | TI ("device related infection*" OR "device acquired infection*" OR "device associated infection*") OR AB ("device related infection*" OR "device acquired infection*" OR "device associated infection*") | 802 |
| 14. | TI ("central line related bloodstream infection*" OR "central line acquired bloodstream infection*" OR "central line associated bloodstream infection*") OR AB ("central line related bloodstream infection*" OR "central line acquired bloodstream infection*" OR "central line associated bloodstream infection*") | 577 |
| 15. | TI (CLABSI OR CRBSI) OR AB (CLABSI OR CRBSI) | 720 |
| 16. | TI bacter#emia OR AB bacter#emia | 23,489 |
| 17. | (MH "Bacteremia") | 19,871 |
| 18. | (MH "Methicillin-Resistant Staphylococcus aureus") | 8,922 |
| 19. | TI "met#icillin resistant staph*" OR AB "met#icillin resistant staph*" | 18,105 |
| 20. | TI MRSA OR AB MRSA | 16,565 |
| 21. | (MH "Clostridium difficile") OR (MH "Clostridium Infections") OR (MH "Enterocolitis, Pseudomembranous") | 13,669 |
| 22. | TI "clostridium difficile" OR AB "clostridium difficile" | 9,937 |
| 23. | TI "c. diff*" OR AB "c. diff*" | 6,478 |
| 24. | TI (CDI OR CDAD) OR AB (CDI OR CDAD) | 4,417 |
| 25. | TI ("extended-spectrum beta-lactamase" OR "extended-spectrum β-lactamase") OR AB ("extended-spectrum beta-lactamase" OR "extended-spectrum β-lactamase") | 4,588 |
| 26. | TI ESBL OR AB ESBL | 4,519 |
| 27. | TI (enterobacter OR enterobacteriaceae) OR AB (enterobacter OR enterobacteriaceae) | 20,093 |
| 28. | (MH "Enterobacter+") | 6,045 |
| 29. | (MH "Enterobacteriaceae Infections+") OR (MH "Enterobacteriaceae") | 100,957 |
| 30. | TI (e.coli OR ecoli) OR AB (e.coli OR ecoli) | 3,469 |
| 31. | (MH "Escherichia coli+") OR (MH "Escherichia coli Infections+") | 256,160 |
| 32. | (MH "Klebsiella Infections+") OR (MH "Klebsiella+") | 18,025 |
| 33. | TI klebsiella OR AB klebsiella | 23,009 |
| 34. | (MH "Citrobacter+") | 2,017 |
| 35. | TI citrobacter OR AB citrobacter | 4,026 |
| 36. | (MH "Serratia+") OR (MH "Serratia Infections") | 6,000 |
| 37. | TI serratia OR AB serratia | 7,868 |
| 38. | (MH "Proteus+") OR (MH "Proteus Infections") | 10,255 |
| 39. | TI (proteus OR proteeae) OR AB (proteus OR proteeae) | 10,978 |
| 40. | TI acinetobacter OR AB acinetobacter | 11,392 |
| 41. | (MH "Acinetobacter+") OR (MH "Acinetobacter Infections") | 7,309 |
| 42. | TI ( "multi resistant" OR "multiresistant" OR "multidrug resistant" OR "multi drug resistant" ) OR AB ( "multi resistant" OR "multiresistant" OR "multidrug resistant" OR "multi drug resistant") | 26,149 |
| 43. | TI ( MDRO OR MRAB ) OR AB ( MDRO OR MRAB) | 162 |
| 44. | TI "carbapenem producing" OR AB "carbapenem producing" | 11 |
| 45. | TI "carbapenem resistant" OR AB "carbapenem resistant" | 1,843 |
| 46. | (MH "Pneumonia, Ventilator-Associated") | 2,300 |
| 47. | TI ("ventilator acquired pneumonia" OR "ventilator associated pneumonia") OR AB ("ventilator acquired pneumonia" OR "ventilator associated pneumonia") | 3,773 |
| 48. | TI VAP OR AB VAP | 2,971 |
| 49. | (MH "Surgical Wound Infection") | 30,383 |
| 50. | TI "surgical wound infection*" OR AB "surgical wound infection*" | 1,116 |
| 51. | TI "surgical site infection*" OR AB "surgical site infection*" | 5,433 |
| 52. | TI SSI OR AB SSI | 3,935 |
| 53. | (MH "Vancomycin-Resistant Enterococci") | 105 |
| 54. | TI "vancomycin resistant enterococc*" OR AB "vancomycin resistant enterococc*" | 3,701 |
| 55. | TI VRE OR AB VRE | 2,172 |

**COMPONENTS OF IPC PROGRAMS**

| 56. | (MH "Hand Hygiene") | 504 |
| --- | --- | --- |
| 57. | TI "hand hygiene" OR AB "hand hygiene" | 2,667 |
| 58. | (MH "Hand Disinfection") | 4,744 |
| 59. | TI (handwashing OR "hand washing") AB (handwashing OR "hand washing") | 431 |
| 60. | TI (handrub* OR "hand rub*") OR AB (handrub* OR "hand rub*") | 595 |
| 61. | TI "hand sanit*" OR AB "hand sanit*" | 249 |
| 62. | TI "hand clean*" OR AB "hand clean*" | 149 |
| 63. | TI ("5 moments" OR "five moments") OR AB ("5 moments" OR "five moments") | 88 |
| 64. | TI ("6 step*" OR "six step*") OR AB ("6 step*" OR "six step*") | 1,758 |
| 65. | TI "alcohol based hand rub" OR AB "alcohol based hand rub" | 170 |
| 66. | TI "alcohol based hand sanit*" OR AB "alcohol based hand sanit*" | 81 |
| 67. | TI "hand gel" OR AB "hand gel" | 68 |
| 68. | TI (ABHR OR ABHS) OR AB (ABHR OR ABHS) | 72 |
| 69. | (MH "Patient Participation") | 19,472 |
| 70. | TI empowerment OR AB empowerment | 7,338 |
| 71. | (MH "Education") OR (MH "Competency-Based Education") OR (MH "Education, Professional+") OR (MH "Inservice Training+") OR (MH "Models, Educational")) | 292,303 |
| 72. | TI education OR AB education | 317,966 |
| 73. | TI training OR AB training | 279,892 |
| 74. | TI "staff development" OR AB "staff development" | 1,514 |
| 75. | (MH "Teaching") | 43,843 |
| 76. | TI teaching OR AB teaching | 107,657 |
| 77. | TI program* OR AB program* | 637,014 |
| 78. | TI course* OR AB course* | 500,893 |
| 79. | TI (workshop* OR seminar* OR module*) OR AB (workshop* OR seminar* OR module*) | 78,540 |
| 80. | TI care N1 bundle* OR AB care N1 bundle* | 421 |
| 81. | TI campaign* OR AB campaign* | 29,609 |
| 82. | TI (multimodal OR "multi modal") OR AB (multimodal OR "multi modal") | 20,577 |
| 83. | (MH "Infection Control") OR (MH "Antisepsis+") | 24,133 |
| 84. | TI ("infection control" OR "infection prevention") OR AB ("infection control" OR "infection prevention") | 16,869 |
| 85. | TI (IC OR IPC) AB (IC OR IPC) | 992 |
| 86. | (MH "Public Health") OR (MH "Preventive Medicine") | 75,823 |
| 87. | (MH "Population Surveillance") OR (MH "Public Health Surveillance") OR (MH "Sentinel Surveillance") | 54,691 |
| 88. | TI surveillance OR AB surveillance | 121,681 |
| 89. | (MH "Clinical Audit") | 1,008 |
| 90. | TI audit* OR AB audit* | 114,916 |
| 91. | (MH "Feedback") OR (MH "Feedback, Psychological") | 29,522 |
| 92. | TI feedback OR AB feedback | 96,870 |
| 93. | (MH "Epidemiology") OR (MH "Molecular Epidemiology") | 21,296 |
| 94. | TI epidemiolog* OR AB epidemiolog* | 289,732 |
| 95. | TI "universal precaution*" OR AB "universal precaution*" | 1,104 |
| 96. | TI "standard precaution*" OR AB "standard precaution*" | 476 |
| 97. | TI SICP OR AB SICP | 34 |
| 98. | TI "transmission precaution*" OR AB "transmission precaution*" | 15 |
| 99. | TI "transmission based precaution*" OR AB "transmission based precaution*" | 45 |
| 100. | TI TBP OR AB TBP | 3,468 |
| 101. | TI "contact precaution*" OR AB "contact precaution*" | 411 |
| 102. | TI "barrier precaution*" OR AB "barrier precaution*" | 331 |
| 103. | (MH "Universal Precautions") | 1,527 |
| 104. | TI (isolate OR isolation OR isolating) OR AB (isolate OR isolation OR isolating) | 285,530 |
| 105. | (MH "Hospitals, Isolation") | 78 |
| 106. | (MH "Patient Isolation") | 3,352 |
| 107. | TI "patient placement" OR AB "patient placement" | 109 |
| 108. | TI ("single room*" OR "side room*") OR AB ("single room*" OR "side room*") | 493 |
| 109. | TI cohorting OR AB cohorting | 322 |
| 110. | (MH "Patients' Rooms") | 2,190 |
| 111. | TI (spac* W3 bed OR "bed spac*") OR AB ( spac* W3 bed OR "bed spac*") | 225 |
| 112. | TI "cough etiquette" OR AB "cough etiquette" | 24 |
| 113. | TI "respiratory hygiene" OR AB "respiratory hygiene" | 42 |
| 114. | TI "personal protective equipment" OR AB "personal protective equipment" | 1,553 |
| 115. | (MH "Gloves, Protective") OR (MH "Gloves, Surgical") | 4,398 |
| 116. | TI glove* OR AB glove* | 8,384 |
| 117. | TI gown* OR AB gown* | 955 |
| 118. | (MH "Masks") | 3,653 |
| 119. | TI (“face mask*” OR “surgical mask*”) OR AB (“face mask*” OR “surgical mask*”) | 2,828 |
| 120. | TI apron* OR AB apron* | 739 |
| 121. | TI "overgown*" OR AB "overgown*" | 4 |
| 122. | (MH "Eye Protective Devices") | 1,587 |
| 123. | TI (goggle* OR eyewear OR visor*) OR AB (goggle* OR eyewear OR visor*) | 1,717 |
| 124. | (MH "Disposable Equipment") | 4,735 |
| 125. | TI ("disposable equipment" OR "single use equipment") OR AB ("disposable equipment" OR "single use equipment") | 182 |
| 126. | TI "sanit* equipment" OR AB "sanit* equipment" | 25 |
| 127. | (MH "Sterilization+") | 26,553 |
| 128. | TI disinfect* OR AB disinfect* | 21,152 |
| 129. | TI decontamination OR AB decontamination | 7,632 |
| 130. | (MH "Decontamination") | 3,827 |
| 131. | (MH "Waste Management") OR (MH "Waste Disposal Facilities") | 5,879 |
| 132. | TI "waste management" OR AB "waste management" | 2,864 |
| 133. | (MH "Medical Waste Disposal") | 1,884 |
| 134. | TI ("medical waste disposal" OR "clinical waste disposal") OR AB ("medical waste disposal" OR "clinical waste disposal") | 59 |
| 135. | (MH "Quality Improvement") | 9,657 |
| 136. | TI "quality improvement" OR AB "quality improvement" | 20,050 |
| 137. | TI "quality management" OR AB "quality management" | 4,870 |
| 138. | (MH "Total Quality Management") | 12,004 |
| 139. | (MH "Leadership") | 33,106 |
| 140. | TI leadership OR AB leadership | 24,164 |
| 141. | TI (strategy OR strategies OR strategic) OR AB (strategy OR strategies OR strategic) | 696,691 |
| 142. | TI (policy OR policies) OR AB (policy OR policies) | 166,989 |
| 143. | (MH "Policy") OR (MH "Organizational Policy") OR (MH "Public Policy") OR (MH "Health Policy") OR (MH "Health Care Reform") | 121,263 |
| 144. | TI "evidence based practice" OR AB "evidence based practice" | 6,871 |
| 145. | (MH "Evidence-Based Practice+") | 68,368 |
| 146. | (MH "Benchmarking") | 10,902 |
| 147. | TI (benchmarking OR "bench marking") OR AB (benchmarking OR "bench marking") | 4,804 |
| 148. | TI "target setting" OR AB "target setting" | 131 |
| 149. | (MH "Goals") | 13,001 |
| 150. | TI "goal setting" OR AB "goal setting" | 2,318 |
| 151. | (MH "Patient Safety") | 8,641 |
| 152. | TI "patient safety" OR AB "patient safety" | 17,924 |
| 153. | (MH "Health Resources") OR (MH "Health Manpower") | 21,134 |
| 154. | TI resourcing OR AB resourcing | 437 |
| 155. | (MH "Personnel Staffing and Scheduling") | 14,962 |
| 156. | TI (staff OR staffing) OR AB (staff OR staffing) | 118,189 |
| 157. | TI manpower OR AB manpower | 6,187 |
| 158. | TI workforce OR AB workforce | 14,779 |
| 159. | TI funding OR AB funding | 28,391 |
| 160. | (MH "Training Support+") | 10,704 |
| 161. | (MH "Intervention Studies") | 0 |
| 162. | TI intervention* OR AB intervention* | 599,479 |

**NATIONAL INTERVENTIONS**

| 163. | TI region* OR AB region* | 1,247,545 |
| --- | --- | --- |
| 164. | TI (state OR states OR statewide) NOT TI "state of" OR AB (state OR states OR statewide) NOT AB "state of" | 335,895 |
| 165. | (MH "Government") OR (MH "Federal Government") OR (MH "United States Dept. of Health and Human Services+") OR (MH "National Institutes of Health (U.S.)") OR (MH "United States Public Health Service") OR (MH "Centers for Disease Control and Prevention (U.S.)") OR (MH "United States Agency for Healthcare Research and Quality") OR (MH "United States Health Resources and Services Administration") OR (MH "Government Agencies") OR (MH "Local Government") OR (MH "State Government") OR (MH "Government Programs") | 116,075 |
| 166. | TI government* OR AB government* | 67,986 |
| 167. | TI federal OR AB federal | 37,208 |
| 168. | TI (countrywide OR "country wide") OR AB (countrywide OR "country wide") | 1,281 |
| 169. | TI (province OR provinces OR provincial) OR AB (province OR provinces OR provincial) | 62,249 |
| 170. | TI (nation OR nations OR national OR nationwide OR "nation wide") OR AB (nation OR nations OR national OR nationwide OR "nation wide") | 376,213 |
| 171. | (MH "National Health Programs") OR MH "Regional Health Planning") OR (MH "Regional Medical Programs") OR (MH "State Health Plans") | 40,259 |

**OUTCOMES**

| 172. | (MH "Mortality+") | 304,241 |
| --- | --- | --- |
| 173. | TI mortality OR AB mortality | 533,289 |
| 174. | TI fatalit* AB fatalit* | 1,854 |
| 175. | (MH "Morbidity") OR (MH "Basic Reproduction Number") | 26,126 |
| 176. | TI morbidity OR AB morbidity | 275,361 |
| 177. | (MH "Length of Stay") | 65,022 |
| 178. | TI "length of stay" OR AB "length of stay" | 34,399 |
| 179. | (MH "Quality-Adjusted Life Years") | 8,023 |
| 180. | TI ("quality adjusted life years" OR "disability adjusted life years") OR AB ("quality adjusted life years" OR "disability adjusted life years") | 5,985 |
| 181. | TI (QALY* OR DALY*) OR AB (QALY* OR DALY*) | 8,103 |
| 182. | (MH "Costs and Cost Analysis+") | 194,005 |
| 183. | TI (cost or costs) OR AB (cost or costs) | 375,194 |
| 184. | TI burden OR AB burden | 114,615 |
| 185. | TI (rate or rates) OR AB (rate or rates) | 2,074,515 |
| 186. | TI incidence OR AB incidence | 570,363 |
| 187. | (MH "Incidence") | 199,568 |
| 188. | TI prevalence OR AB prevalence | 442,601 |
| 189. | (MH "Prevalence") | 213,060 |
| 190. | TI (reduce or reduces or reduced or reduction or reductions) OR AB (reduce or reduces or reduced or reduction or reductions) | 2,263,542 |
| 191. | TI "consumption of alcohol based hand rub" OR AB "consumption of alcohol based hand rub" | 6 |
| 192. | TI "consumption of alcohol based hand sanit*" OR AB "consumption of alcohol based hand sanit*" | 1 |
| 193. | TI "consumption of ABHR" OR AB "consumption of ABHR" | 3 |
| 194. | TI "use of alcohol based hand rub" OR AB "use of alcohol based hand rub" | 37 |
| 195. | TI "use of alcohol based hand sanit*" OR AB "use of alcohol based hand sanit*" | 17 |
| 196. | TI ("use of ABHR" OR "use of ABHS" OR "use of hand gel") OR AB ("use of ABHR" OR "use of ABHS" OR "use of hand gel") | 14 |
| 197. | TI (compliance OR concordance OR adherence) NOT TI (medication OR treatment) OR AB (compliance OR concordance OR adherence) NOT AB (medication OR treatment) | 133,903 |
| 198. | (MH "Guideline Adherence") | 23,698 |
| 199. | TI knowledge OR AB knowledge | 472,203 |
| 200. | (MH "Health Knowledge, Attitudes, Practice") | 80,874 |
| 201. | TI (competence OR competency) OR AB (competence OR competency) | 51,746 |
| 202. | (MH "Professional Competence+") | 92,370 |
| 203. | TI effective* OR AB effective* | 1,401,294 |
| 204. | TI (improve OR improves OR improved OR improvement) OR AB (improve OR improves OR improved OR improvement) | 1,501,514 |
